# Prognostic value of DNA repair based stratification of hepatocellular carcinoma

**DOI:** 10.1038/srep25999

**Published:** 2016-05-13

**Authors:** Zhuo Lin, Shi-Hao Xu, Hai-Qing Wang, Yi-Jing Cai, Li Ying, Mei Song, Yu-Qun Wang, Shan-Jie Du, Ke-Qing Shi, Meng-Tao Zhou

**Affiliations:** 1 Department of Infectious and Liver Diseases, The First Affiliated Hospital of Wenzhou Medical University, Wenzhou 325000, China; 2 Institute of Hepatology, Wenzhou Medical University, Wenzhou, China; 3 Department of Ultrasonography, The First Affiliated Hospital of Wenzhou Medical University, Wenzhou 325000, China; 4 Reproductive Medicine Center, The First Affiliated Hospital of Wenzhou Medical University, Wenzhou 325000, China; 5 Department of Hepatobiliary Surgery, The First Affiliated Hospital of Wenzhou Medical University, Wenzhou, China

## Abstract

Aberrant activation of DNA repair is frequently associated with tumor progression and response to therapy in hepatocellular carcinoma (HCC). Bioinformatics analyses of HCC data in the Cancer Genome Atlas (TCGA) were performed to define DNA repair based molecular classification that could predict the prognosis of patients with HCC. Furthermore, we tested its predictive performance in 120 independent cases. Four molecular subgroups were identified on the basis of coordinate DNA repair cluster (CDRC) comprising 15 genes in TCGA dataset. Increasing expression of CDRC genes were significantly associated with TP53 mutation. High CDRC was significantly correlated with advanced tumor grades, advanced pathological stage and increased vascular invasion rate. Multivariate Cox regression analysis indicated that the molecular subgrouping was an independent prognostic parameter for both overall survival (p = 0.004, hazard ratio (HR): 2.989) and tumor-free survival (p = 0.049, HR: 3.366) in TCGA dataset. Similar results were also obtained by analyzing the independent cohort. These data suggest that distinct dysregulation of DNA repair constituents based molecular classes in HCC would be useful for predicting prognosis and designing clinical trials for targeted therapy.

Hepatocellular carcinoma (HCC) is the leading cause of cancer death worldwid with dismal prognosis^1^. Although surgical resection offers the best chance of cure of HCC, the prognosis after surgery differs considerably among patients, which has hampered both treatment and prognostic prediction. Much work has been devoted to identifying histopathological and biochemical markers or establishing prognostic models for identifying groups of HCC that differ with respect to prognosis^2^. However, HCC, even in the same tissue, is a very heterogeneous disease that differs widely in clinical outcome and in response to therapy^3^. Different molecular defects that can induce similar tumor phenotypes predispose the heterogeneity of HCC^4^. Therefore, genomic information from tumor tissue will refine the prognostic prediction of HCC patients, and facilitate the identification of genetic determinants that are components of the specific regulatory pathways altered in cancers, providing the opportunity to precision medicine^5^.

The application of microarray or high-throughput technologies which rely on thousands of pieces of bio-information and provide an accurate landscape of HCC genetic alterations have enabled researchers to measure the expression of a large number of genes in HCC for identifying tumor subtypes^6^. Gene expression profiling studies in HCC have reported molecular signatures that were associated with prognosis^7^,^8^,^9^. However, most studies that have been conducted to date involved relatively few patients, which was not clear whetner results could be generalized into large clinical populations with the collection of samples at different sites making for variation in study cohort. As such, a coherent molecular explanation for the HCC heterogeneity and prognostic prediction has yet to be reported. Furthermore, laboratory-based functional analysis is an important part of the evaluation for microarray or high-throughput analysis, and it remains unclear how this would be incorporated into routine clinical application of sophisticated and non-uniform gene-expression signatures in HCC prognostic prediction^9^,^10^. Therefore, gene-expression pattern of the specific HCC regulatory pathways which could predict or stratify the prognostic subgroups of HCC needed to be identified^11^,^12^.

DNA repair process is constantly active as it responds to damage in the DNA structure and proceed by several mechanisms, including base excision repair, mismatch excision repair, nucleotide excision repair, homologous recombination^13^. A cell that has accumulated a large amount of DNA damage, or one that no longer effectively repairs damage incurred to its DNA, can enter one of three possible states: senescence, apoptosis and cancerous^14^. Cancer tissues overexpress DNA repair genes and thus develop greater DNA repair capacity than normal tissues^15^. Therefore, multiple DNA damage signals and DNA repair pathways could have a significant impact on prognosis and response to therapy for various cancers^16^.

A thorough understanding of the DNA repair genes expression pattern in tumors would be of utmost importance in HCC prognostic prediction and improving therapy and in achieving the best therapeutic response^17^. In the present study, we investigated the possibility that the expression pattern of the HCC associated DNA repair genes obtained at diagnosis would permit the identification of distinct subclasses of HCC patients with different prognosis in the Cancer Genome Atlas (TCGA) dataset. Furthermore, we tested the expression pattern of co-regulated cluster of DNA repair genes at both protein and mRNA level to explore the relationship with prognosis of HCC patients in another separate cohort.

## Results

### Molecular subgroups based on DNA repair genes in hepatocellular carcinoma

Based on previous studies on molecular networks incorporating DNA repair genes linked with hepatocarcinogenesis or progression in HCC, 59 initial genes were included in the preliminary analyses^13^,^18^,^19^ (Supplementary Table S1). These genes refered to mismatch excision repair (MSH2, MSH3, MSH6, MLH1, PMS2, MLH3), base excision repair (MBD4, TDG, OGG1, NEIL3, APEX1), nucleotide excision repair (XPC, RAD23B, XPA, DDB1, RPA2, ERCC6), homologous recombination (RAD51, XRCC3, RAD52, BRCA1, RAD50, MRE11A, NBN, MUS81), non-homologous end-joining (XRCC6, XRCC5, PRKDC, LIG4, XRCC4), chromatin structure and modification, and DNA polymerases. DNA damage response genes (ATR, CHEK1, CHEK2, TP53, TP53BP1, WRN, ATM) were also included.

**Figure 1:**
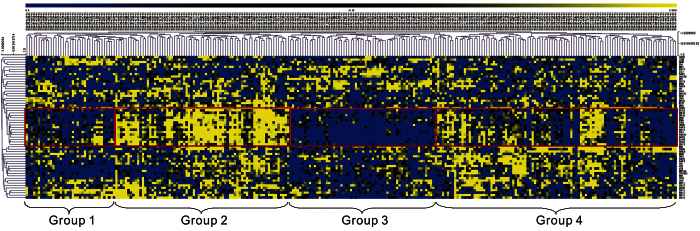
Co-ordinated DNA repair gene expression cluster identified by Two-dimensional hierarchical clustering. Clustering was performed by gene expression (rows) and patients (columns) using the Pearson algorithm. Yellow represents high gene expression, black represents intermediate gene expression and blue represents low gene expression. The red box shows a group of closely associated genes that had a co-ordinated expression pattern across the patient population.

**Figure 2:**
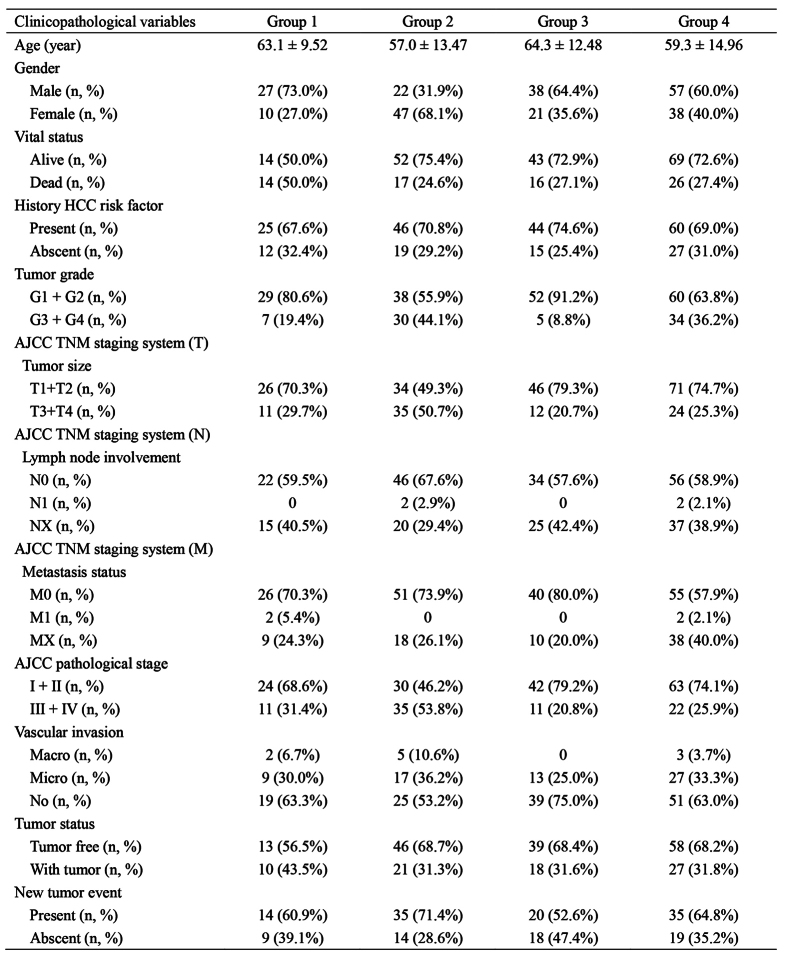
Clinical characteristics of patient groups by coordinate DNA repair cluster.

Unsupervised two-way hierarchical clustering analysis was performed to assess the extent to which gene expression was coordinate or independent across the patient cohort. A 15-gene subset was identified by the visual analysis of the clustering, expression of which was coordinately regulated and formed a clear gene grouping (Fig. 1, Supplementary Table S2). The validity of this gene clustering was confirmed by the gene-tree analysis of the dendrogram. These 15 highly coordinated genes (distance threshold 1.64) associated with some different DNA repair pathways, including mismatch excision repair, base excision repair, homologous recombination, and DNA damage response genes. The degree of correlation among the 15 genes was notable (Supplementary Fig. S1). The genes formed a clearly coordinated block on visual and correlation analysis. Therefore, we proceeded with the 15-gene block and termed this cluster as coordinate DNA repair cluster (CDRC). Four distinct patient groups were delineated by the CDRC (Fig. 1). The patients in the group 2 had the highest CDRC expression (mean expression 1.11), while the lowest in the group 3 (mean expression −0.60). Clinical characteristics of patients in the four groups by CDRC were shown in Fig. 2.

### Molecular determinants of the coordinate DNA repair cluster

Each tumor accumulates numerous damaging mutations, therefore, three major groups of associated alterations, including the CTNNB1 cluster (CTNNB1, TERT, KMT2D, ARID2, APOB, NFE2L2), the AXIN1 cluster (AXIN1, ARID1A, RPS6KA3) and the tumour protein p53 (TP53) cluster (TP53, KEAP1, CCND1, TSC2) were identified by exome sequencing of HCC^20^. The mutational status of three major groups in the cohort was as follows: CTNNB1 mutant 19.0%, KMT2D mutant 7.5%, ARID2 mutant 2.2%, APOB mutant 9.0%, NFE2L2 mutant 1.5%, AXIN1 mutant 3.4%, ARID1A mutant 5.6%, RPS6KA3 mutant 2.2%, TP53 mutant 22.4%, KEAP1 mutant 2.6%, and TSC2 mutant 3.4%. None variant of the TERT or CCND1 was found in this cohort. Table 1 showed the distribution of three major groups of associated alterations in each distinct patient group. The frequency of TP53 mutation was significantly lower in group 3 than other patient groups, group 3 (6.7%) < group 4 (20.2%) < group 1 (34.3%) < group 2 (37.5%), (p < 0.05). Multivariate linear regression analysis indicated that all the CDRC genes expression were significantly associated with TP53 mutation (p < 0.01). There was a strong trend for CTNNB1 mutation to be associated with low CDRC expression. Multivariate analysis revealed that decreased expression of ERCC6, FANCD2, H2AFX, MSH6, RAD51, and XRCC3 were associated with CTNNB1 mutation (p < 0.05, Supplementary Table S3).

**Figure 3:**
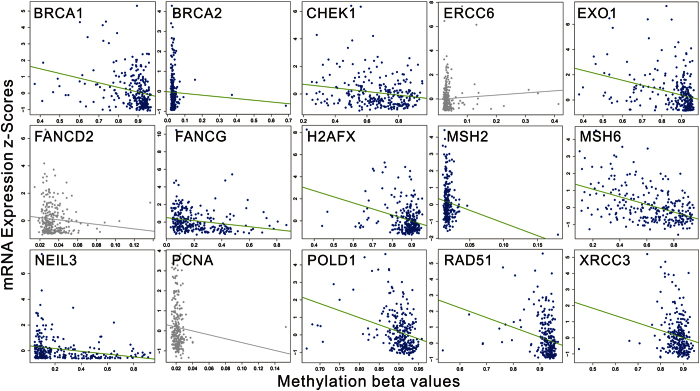
Correlation between methylation beta-values and mRNA expression Z score of the coordinate DNA repair cluster genes. The coloured represents significant negative correlation between methylation beta-values and mRNA expression Z score. The gray represents no correlation.

To determine whether any other molecular characteristics were associated with CDRC expression, we analyzed the correlation of the methylation and copy number status of the CDRC with the gene expression. Interestingly, most CDRC genes had a significant negative correlation between methylation beta-values and mRNA expression Z score (Fig. 3). Furthermore, copy number statuses, including putative copy-number alterations from GISTIC and log2 copy-number values, were significantly positive associated with gene mRNA expression in most CDRC genes (Supplementary Figs S2 and S3).

### Molecular subgroups correlate with clinicopathological variables

Age distribution was closely associated with four DNA repair based molecular subgroups of HCC described herein. Patients in group 2 had lower mean age compared to other CDRC clusters (p < 0.05). The gender distribution of all subtypes was presented in Fig. 2, with some significant differences between subgroups. There was a preponderance of males represented in the group 1 (male:female = 2.7:1), group 3 (male:female = 1.8:1) and group 4 (male:female = 1.5:1) subgroups, whereas in the group 2, males were in the minority (male:female = 0.47:1) (p < 0.001). The history HCC risk factor distribution in each subgroup was similar. Advanced tumor grade (grade G3–G4), advanced AJCC pathological stage (stage III–IV), and vascular invasion were observed in lower frequencies in group 3 compared to the other groups (Supplementary Fig. S4). For each individual aspect of AJCC TNM staging system, the frequencies of T1/2 and no distant metastasis were considerably higher in Group 3; the frequency of no regional lymph node involvement was also relatively lower in Group 3.

### Molecular subgroups correlate with hepatocellular carcinoma progression

**Figure 4:**
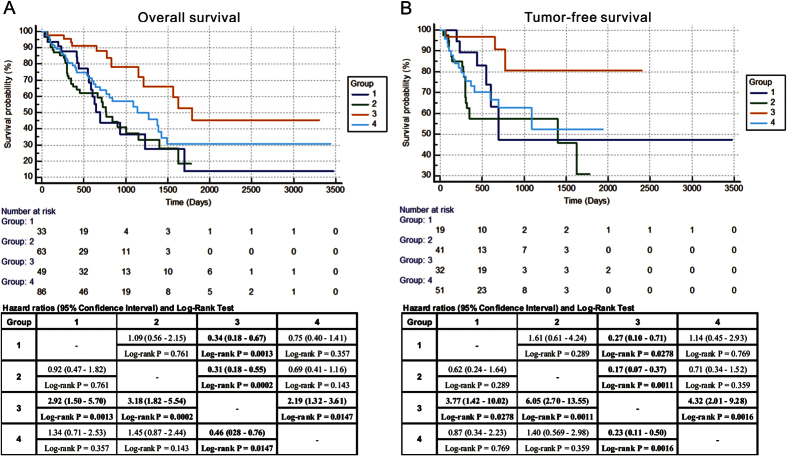
DNA repair molecular classes were correlated with overall survival (**A**) and tumor-free survival (**B**) in HCC patients. Kaplan-Meier survival curves show group 3 was significantly correlated with favorable survival of HCC. *P*-values were calculated by log-rank test. Hazard ratios (95% Confidence Interval) and Log-rank Test were shown in the Tables. HCC, hepatocellular carcinoma.

**Figure 5:**
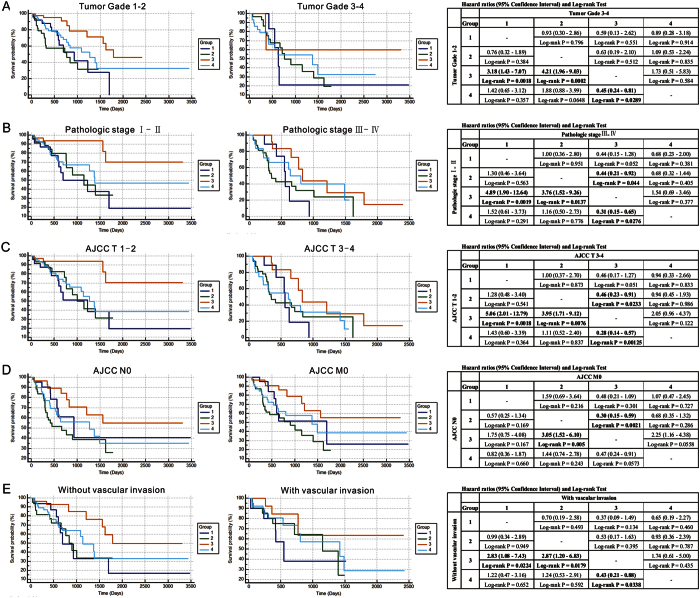
Correlation between DNA repair molecular classes and overall survival in HCC patients was independent of tumor grade, pathological stage and vascular invasion. (**A**) Comparisons of overall survival in DNA repair molecular classes of HCC in early tumor grade (G1–G2) cohort and in advanced tumor grade (G3–G4) cohort. (**B**) Comparisons of overall survival in DNA repair molecular classes of HCC in early pathological stage (I–II) cohort and in advanced pathological stage (III–IV) cohort. (**C**) Comparisons of overall survival in DNA repair molecular classes of HCC in early pathological T classification (T1–T2) cohort and in advanced pathological T classification (T3–T4) cohort. (**D**) Comparisons of overall survival in DNA repair molecular classes of HCC in pathological no local lymph node metastasis cohort and in pathological no metastasis cohort. (**E**) Comparisons of overall survival in DNA repair molecular classes of HCC in patients with or without vascular invasion. P-values were calculated by log-rank test. Hazard ratios (95% Confidence Interval) and Log-rank Test were shown in the Tables. HCC, hepatocellular carcinoma.

Survival analyses for all patients with available outcome data (n = 239) showed remarkable differences in overall survival (OS) and tumor-free survival (TFS) between the molecular subgroups. Patients in group 3 showed a favorable outcome, with 5-year OS rates of ~45% (Fig. 4A) and PFS rates of ~75% (Fig. 4B). Notably, patients comprising this subgroup had the lowest frequency of TP53 mutation, which was consistent with published literatures^21^,^22^. All other subgroups have a more dismal outcome, with 5-year OS rates ranging from 14–30% (Fig. 4A), and TFS rates ranging from 31–51% (Fig. 4B). Although a substantial proportion of patients in group 3 was classified as favorable histology (early tumor grades, early AJCC pathological stages, and without vascular invasion), correlation between molecular subgroups (especially between group 2 and group 3) and OS and TFS rate in HCC patients was independent of tumor grade (Fig. 5A, Supplementary Fig. S5A), AJCC pathological stage (Fig. 5B, Supplementary Fig. S5B), and vascular invasion (Fig. 5E, Supplementary Fig. S5E). Therefore, as one example for the clinical utility of molecular subgrouping over conventional histopathology, molecular classification by CDRC in these instances revealed HCC subgroup with favorable outcome that would not be yielded by histopathological analysis.

Examining other clinical variables for subgroup-specific prognostic value showed that OS and TFS rate in HCC patients between group 2 and group 3 were independent of gender, age, and HCC history risk factors (Supplementary Figs S6 and S7). Although, TP53 mutation showed no prognostic value in the present series, highly significant differences in OS and TFS between group 2 and group 3 were also seen in the patients without TP53 mutation (Supplementary Fig. S8).

According to the results above, a multivariate Cox regression analysis was performed to assess whether CDRC molecular subgrouping was independent prognostic parameters for HCC in group 2 and group 3. Results showed that in group 2 and group 3, CDRC molecular subgrouping was the independent prognostic parameter for both OS (hazard ratio (HR): 2.989, 95% confidence interval (CI) 1.431–6.243, P = 0.004) and TFS (HR: 3.366, 95% CI 1.053–11.889, P = 0.049) (Table 2). Pathological stage and T classification were also the independent prognostic parameter for OS and TFS, respectively. Furthermore, the performance of CDRC clusters for identifying the HCC subgroups was validated in our own cohort. Due to the consistent expression of the genes in CDRC clusters (Supplementary Fig. S1), we characterized the expression pattern of MSH2 which is the component of the post-replicative DNA mismatch repair system^23^ in HCC tissues and assessed the clinical significance of MSH2 expression in HCC patients from an independent cohort. One hundred and twenty patients were enrolled with m ean follow-up of 612.0 ± 230.2 days. The clinicopathological characteristics of the patients were summarized in Supplementary Table S4.

**Figure 6:**
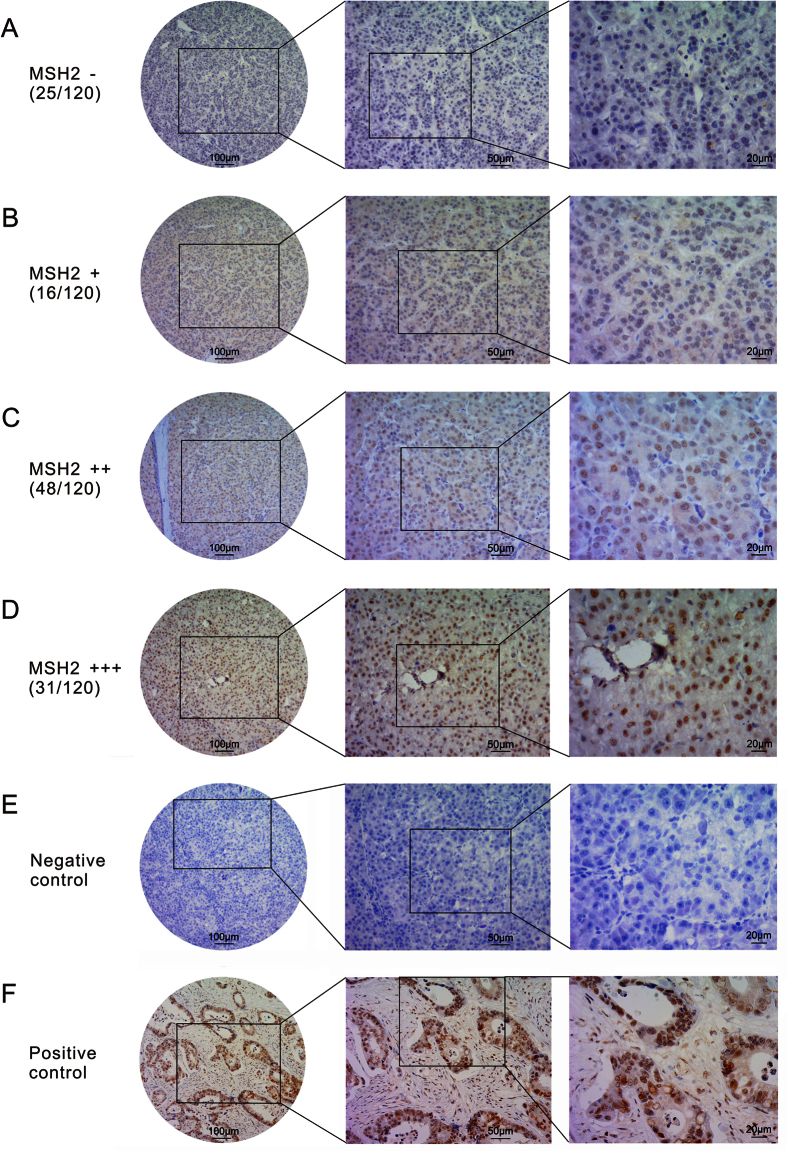
MSH2 expression in HCC tissue samples by immunochemistry. (**A**–**F**), Representative images of MSH2 expression in HCC and control. (**A**) MSH2, scored as (−); (**B**) MSH2, scored as (+); (**C**) MSH2, scored as (++); (**D**) MSH2, scored as (+++); (**E**) negative control; (**F**) positive control, MSH2 expression in colonic adenocarcinoma, scored as (+++).

**Figure 7:**
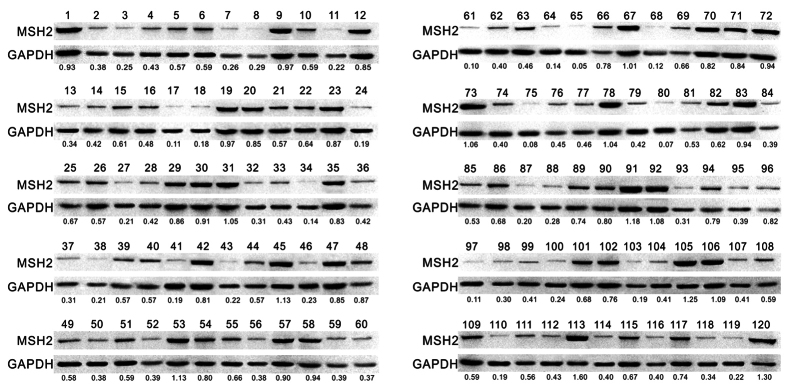
Western blotting analysis of MSH2 protein expression in HCC tissues. The low MSH2 expression group was defined: protein level < 0.4 (n = 41); the moderate MSH2 expression group was defined: 0.4 ≤ protein level < 0.8 (n = 48); the high MSH2 expression group was defined: protein level ≥ 0.8 (n = 31). MSH2 protein level was normalized by GAPDH. HCC, hepatocellular carcinoma.

MSH2 protein expression was evaluated by immunohistochemical staining. According to the MSH2 expression pattern by immunohistochemical staining, HCC tissues was divided into low (n = 41), moderate (n = 48) and high (n = 31) expression groups (Fig. 6). The characteristics of patient groups by MSH2 expression pattern were shown Table 3. MSH2 protein level analyzed by Western blot (Fig. 7) was consistent with that by immunochemistry. MSH2 mRNA expression levels were examined using real-time quantitative PCR in the same tumor tissues. The results demonstrated that MSH2 mRNA expression pattern was similar to protein level (Supplementary Fig. S9). Furthermore, MSH2 expression pattern (between low expression group and high expression group) in HCC tissues was closely correlated with pathological stage (P < 0.001) and T classification (P < 0.001). No significant associations were observed between MSH2 expression pattern (between low expression group and high expression group) and age, gender, tumor size, tumor grade and vascular invasion. As shown in Supplementary Fig. S10, low MSH2 expression pattern was associated with favorable OS (P < 0.001). Kaplan-Meier analyses showed that mortality was lower in HCC patients with lower MSH2 expression, which was independent of pathological stage, pathologic T classification, tumor grade, vascular invasion, gender, and age (Supplementary Fig. S10). Multivariate Cox regression analyses showed that MSH2 expression pattern (between high expression group and low expression group) could be useful as an independent predictor for the prognosis in HCC patients (HR: 3.375, 95% CI 1.483–7.681, P = 0.004, Supplementary Table S5).

## Discussion

Many genetic and epigenetic disruptions in HCC indicate that it is characterized by remarkable molecular heterogeneity^24^. Gene expression profiles of HCC could define the molecular characteristics of the tumors and show a good prognostic value^25^,^26^,^27^,^28^. However, common laboratory practices can not address the research needs of molecular biology. To focus on HCC specific pathway molecular-stratification, cluster analysis was used for molecular stratification based on DNA repair gene expression patterns in TCGA. In current study, we identified a group of 15 tightly co-regulated DNA repair-related genes termed as CDRC, and used this to assess differences in the prognosis of HCC patients. Four molecular subtypes of HCC with distinct progression were classified by CDRC expression pattern.

The relationship between CDRC expression pattern and clinicopathologic parameters was evaluated. The results indicated that increased DNA repair capacity was correlated with poor tumor differentiation and poor survival^29^,^30^, which was also validated in the independent cohort. However, interestingly, intrinsic HCC subtypes by CDRC were not contingent on tumour stage or other single clinicopathologic parameter. The subtypes differed significantly with respect to several biological and clinical parameters associated with different tumor biologic behavior. Furthermore, the major finding of the current study was that mortality in patients could be predicted accurately using our CDRC based molecular groups, especially between group 2 and group 3. Of note, expression of MSH2, as well as other DNA repair genes^31^,^32^, in tumor tissues has been used in many investigations, revealing that DNA repair capacity could be a potential predictor of prognosis in some human malignancies^33^,^34^. Down-regulation of MSH2 expression by Hsp90 inhibitor could enhance pemetrexed-induced cytotoxicity in human non-small-cell lung cancer cells^35^. Inhibition of p38 MAPK-dependent MSH2 expression by metformin could enhance gefitinib-induced cytotoxicity in human squamous lung cancer cells^36^. EXO1 up-regulated expression could protect ovarian cancer cells from cisplatin-mediated apoptosis, and attenuating EXO1 expression by small interfering RNA could augment the chemotherapy efficacy against ovarian cancer^37^. Therefore, we speculated that prognosis might be an intrinsic property of the HCC classified by CDRC clusters.

DNA repair genes affect cell proliferation or survival indirectly by influencing the ability of the organism to repair nonlethal damage in other genes, including protooncogenes, tumor suppressor genes, and genes that regulate apoptosis^38^,^39^. By removing DNA lesions in tumors, increased DNA repair is one of major mechanisms for development of resistance to therapy, which affects patient survival. Therefore, knowledge of DNA protein expression patterns in cancerous tissues may help guide development of therapeutic strategies and treatments for cancers, and furthermore predict the response and survival. Numerous inhibitors of DNA repair which may help selectively kill tumors have been developed and are being tested in clinical trials. In our study, CDRC refers to mismatch excision repair, base excision repair, nucleotide excision repair, and homologous recombination, which could reflect DNA repair capacity^40^. Therefore, CDRC clusters may potentially lead to personalized therapy for HCC patients^41^.

In addition, we investigated the mutational status of three major groups (CTNNB1 cluster, AXIN1 cluster and TP53 cluster) associated with CDRC expression in HCC. TP53 mutant tumors which had a high mutational burden were associated with high CDRC expression, with only 6.7% of cancers in the group exhibiting very low expression (group 3) being TP53 mutant. Genomic instability could be enhanced by TP53 mutant, which might lead to an increase DNA repair capacity. Furthermore, convincing evidence show that TP53 mutant proteins play an important gain-of-function role in promoting invasion and metastasis of tumors. Recent studies have indicated that genome-wide and cell type-specific alterations in miRNA expression during DNA damage response could be regulated by TP53^42^,^43^. In addition, TP53 mutation could affected the prognosis for patients with HCC in the total population from the TCGA dataset (data not shown), which was consistent with those from previous studies^21^,^44^. However, molecular classification by CDRC was the independent prognostic factor of HCC after adjusting TP53 mutation. CTNNB1 is a target for mutations when mismatch repair is impaired^45^. CTNNB1 mutation was significantly associated with lower CDRC expression, which designated a subset of low-grade, low-stage HCC, and hence more favorable prognosis^46^. Our data complemented previous studies demonstrating widespread abnormalities of DNA repair response in mutational status of three major groups in HCC. Our findings indicated that methylation and copy number status of the CDRC also played an important role to regulate the gene expression.

Molecular characterization of HCC based on DNA repair gene expression would improve the prediction of the clinical outcome of HCC patients and selection of treatments for specific molecular subtypes of HCC. Nevertheless, our findings needed to be validated and further refined in a large prospective patient cohort. Furthermore, the efficacy of adjuvant therapies for HCC, such as chemotherapy, and/or molecular targeted therapies could be assessed in the context of specific molecular subgroups, as the response to different therapy modalities would likely differ.

## Materials and Methods

### Identification of initial DNA repair genes in HCC

Human DNA repair genes or genes in DNA repair pathways were extracted as initial genes^13^,^18^,^19^. Molecular network pathway analysis was used for expanding the gene list, by which further molecules and genes with known genetic, pathway and functional associations with our initial genes could be revealed. Finally, the genes that were relevant to the HCC were screened for inclusion into our final gene list (Supplementary Table S1).

### Expression datasets preparation

HCC TCGA dataset containing Normalized Agilent microarray and RNAseq z-score data, copy-number status generated by GISTIC, log2 copy-number value, and methylation (HM450) beta-values with the most anti-correlated with expression for each initial gene, and mutation data in three major groups: the CTNNB1 cluster (CTNNB1, TERT, MLL2, ARID2, APOB, NFE2L2), the AXIN1 cluster (AXIN1, ARID1A, RPS6KA3) and the TP53 cluster (TP53, KEAP1, CCND1, TSC2) identified by exome sequencing of HCC were extracted^20^. The mRNA expression data was generated using the Illumina HiSeq 2000 RNA Sequencing platform and were normalized to sample medians as previously described. mRNA expression data for each initial gene was downloaded as z-scores from the cBioPortal (http://www.cbioportal.org/)^47^. Copy-number data for HCC samples were generated from array comparative genomic hybridization data acquired using the Affymetrix Genome-Wide Human SNP Array 6.0 platform. Raw data was analyzed using the GISTIC2 method to generate gene-level copy-number calls and downloaded from Memorial Sloan-Kettering Cancer Center’s cBioPortal for Cancer Genomics (http://www.cbioportal.org/). GISTIC2-generated copy-number estimates (log 2 -transformed values, not thresholded) were downloaded from the UCSC Cancer Browser (https://genome-cancer.ucsc.edu). Methylation data was generated using Illumina Infinium Human DNA Methylation 450 platform and downloaded as beta-values from the cBioPortal (http://www.cbioportal.org/)^47^. The genes would be excluded from the analysis, once expression data were unavailable (n = 268, up to June 1st, 2015). The full clinical dataset, including age, gender, the American Joint Committee on Cancer staging system (also be called AJCC TNM staging system), tumor status, vital status, new tumor eventrisk, risk factors, and vascular invasion, were downloaded (up to June 1st, 2015) from the TCGA portal (https://tcga-data.nci.nih.gov/tcga/) and tabulated with genetic data.

### Clinical tissue samples

A prospective cohort of 120 patients with HCC was recruited from the First Affiliated Hospital of Wenzhou Medical University. These patients were diagnosed with HCC between January, 2013 and December, 2014. Patients were excluded if they had a diagnosis of a concurrent cancer or cancers metastatic to the liver. Clinical information of each patient including details of pathology and outcomes with a regularly follow up was collected. The histology and clinical stages were classified according to the seventh edition of the American Joint Committee on Cancer (AJCC) staging system. The tumor grade 1–4 of HCC in pathology diagnosis is equivalent to well-differentiated, moderately-differentiated, poorly differentiated, or undifferentiated, respectively, under microscope (Supplementary Fig. 11). Grade 1 or well-differentiated: Cells appear normal and are not growing rapidly. Grade 2 or moderately-differentiated: Cells appear slightly different than normal. Grade 3 or poorly differentiated: Cells appear abnormal and tend to grow and spread more aggressively. Grade 4 or undifferentiated: features are not significantly distinguishing to make it look any different from undifferentiated cancers which occur in other organs. Vascular invasion is identified either as macroscopic, when the invasion of the vessel is visible on gross examination, or as microscopic, when the invasion is visible only on microscopy. Microscopic vascular invasion was defined as tumoral cells within a vascular space lined by endothelium that was visible only on microscopy, and was assessed by several sections of non-tumoral hepatic parenchyma 1 cm away from the tumor^48^. The cases of HCC were selected in this study only if clinical data were available. The follow-up time was calculated from the date of surgery to the date of death, or the last known follow-up. None of them had received radiotherapy, chemotherapy, hormone therapy or other related anti-tumor therapies before surgery. Research ethics approval for this project was granted from the First Affiliated Hospital of Wenzhou Medical University, and written informed consent was obtained from all patients or their guardians for the use of the biospecimens for research purposes, which were carried out in accordance with the approved guideline “Use of experimental animals and human subjects”. The samples were frozen and stored in liquid nitrogen immediately after surgically resected.

### Immunochemistry

Immunohistochemical staining of MSH2 was performed on the 120 HCC sections. The sections were blocked in 3% hydrogen peroxide solution for 10 min at room temperature and then incubated with the MSH2 primary antibody (1:1000, Abcam, Cambridge, UK) overnight at 4 °C. A negative control was performed by replacing the primary antibody with PBS. The sections were then incubated with a horseradish peroxidase labeled secondary antibody (1:1000, Abcam, Cambridge, UK) at room temperature for 120 min. Finally, the signal was developed for visualization with diaminobenzidine and the sections were counterstained with hematoxylin. MSH2 expression score was conducted according to the percent of positive cells: 0–5% scored 0; 6–35% scored 1; 36–70% scored 2; more than 70% scored 3 and staining intensity: no staining scored 0, weakly staining scored 1, moderately staining scored 2 and strongly staining scored 3. The final score was determined using the ratio of positive cell score × staining intensity score as follows: “−” for a score of 0–1, “+” for a score of 2–3, “++” for a score of 4–6 and “+++” for a score of >6. Low expression was defined as a total score <4, moderate expression with a total score ≥ 4 and ≤6, and high expression with a total score > 6.

### RNA extraction and real-time quantitative PCR

Total RNA was extracted from a section of fresh frozen tumor tissue using the RNeasy Mini Kit (Qiagen, Hilden, DE). Reverse transcription and PCR were executed using the RevertAid First Strand cDNA Synthesis kit (Thermo, Waltham, USA) and Power SYBR Green PCR kit (Applied Biosystems, Carlsbad, USA) according to the manufacturer’s instructions. Quantitative real-time PCR was performed using a 7500 Real-time PCR system (Applied Biosystems, Carlsbad, USA). Primer sequences for MSH2 detection were as follows, forward: 5′-AGAGACAGGTTGGAGTTGG-3′; reverse: 5′-CGGGTAAAACACATTCCTT-3′. The relative expression level of MSH2 was determined using the 2^−∆∆Ct^ method and normalized to β-actin.

### Western blotting

Total protein was extracted using a RIPA lysis buffer (Fdbio science, Hangzhou, China). The proteins were then separated by SDS-PAGE and transferred onto polyvinylidene fluoride membrane. The membrane was blocked with 5% nonfat dried milk and incubated with MSH2 primary antibody (1:5000, Abcam, Cambridge, UK) and goat anti-rabbit secondary antibody (1:10000, Abcam, Cambridge, UK). Proteins were detected by using enhanced chemiluminescence method and imaged by GelDoc™ XR+ system (Bio-Rad, Hercules, USA). GAPDH (1:5000, Abcam, Cambridge, UK) was chosen as an internal control and the expression level of MSH2 protein was normalized by GAPDH.

### Statistical analysis

Unsupervised two-way hierarchical clustering of HCC samples and initial genes expression data was performed with Multiexperiment Viewer (MeV) 4.9.0 (Dana-Farber Cancer Institute, Boston, MA, USA), designed to allow the analysis of microarray data to identify patterns of gene expression and differentially expressed genes. Heatmap view of a diagram will come up that looks like lots of tiny blue and yellow boxes. Each row represents a specific gene, whereas each column represents each patient. Pearson correlation was used as the distance metric for hierarchical clustering. The number of patient and gene clusters can be adjusted by varying sample-tree or gene-tree distance-thresholds and visual analysis. The lower the distance range, the more clusters there will be since a shorter distance between similar genes means more groups. Gene cluster which could identify similar patients would be established. Normality of distributions was confirmed with the Anderson–Darling test. The correlations in gene expression within the main clusters and correlations of genes expression with their copy-number or methylation status were investigated by Pearson Coefficient of determination (R^2^) values using R software (v3.1.2). Multivariate linear regression models were used to analyze associations between genes expression with the mutations in three major groups: the CTNNB1 cluster (CTNNB1, TERT, MLL2, ARID2, APOB, NFE2L2), the AXIN1 cluster (AXIN1, ARID1A, RPS6KA3) and the TP53 cluster (TP53, KEAP1, CCND1, TSC2). An independent two-sample t-test was used for comparison of continuous variables for normally distributed data, and Mann–Whitney U tests for non-normally distributed data. The χ2-test was used for comparison of categorical variables, if any groups contained less than 5, the Fisher’s Exact test was used in preference. Survival curves were evaluated using the Kaplan-Meier method, and differences between survival curves were tested by the log-rank test in the different groups. Cox proportional hazards regression model was used to examine univariate and multivariate hazard ratios for the prognostic parameters, including molecular groups and clinicopathological variables that were dichotomized. Only significantly different variables in univariate analysis were entered into the next multivariate analysis. Furthermore, the performance of DNA repair genes clusters for identifying the HCC subgroups was validated in our own cohort. The deceased patients were excluded in the survival analysis due to overall survival more than 5 years; patients who died or were lost to follow-up within 30 days were also excluded. The analyses were carried out using the SPSS Statistics 21.0 (SPSS Inc., an IBM Company) and MedCalc version 14.8 (MedCalc, Mariakerke, Belgium). A 2-tailed P value < 0.05 was considered to be statistically significant.

## Additional Information

**How to cite this article**: Lin, Z. *et al*. Prognostic value of DNA repair based stratification of hepatocellular carcinoma. *Sci. Rep.* **6**, 25999; doi: 10.1038/srep25999 (2016).

## Supplementary Material

Supplementary Information

## Figures and Tables

**Table 1:** Molecular characteristics of patient groups by coordinate DNA repair cluster.

**Molecular parameter**	**Group 1**	**Group 2**	**Group 3**	**Group 4**
Percentage of patients	37 (13.8%)	72 (26.9%)	60 (22.4%)	99 (36.9%)
CDRC copy number status*				
homozygous deletion	0	3 (0.4%)	0	6 (0.5%)
heemizygous deletion	111 (20.6%)	243 (28.9%)	0	221 (19.0%)
neutral/no change	335 (62.0%)	415 (49.4%)	561 (79.2%)	729 (63.6%)
gain	78 (14.4%)	165 (19.6%)	138 (19.5%)	198 (17.0%)
high-level amplification	16 (3.0%)	14 (1.7%)	9 (1.3%)	10 (0.9%)
CTNNB1 mutation cluster&				
CTNNB1	5 (13.5%)	12 (16.7%)	16 (26.7%)	17 (17.2%)
KMT2D	4 (10.8%)	6 (8.3%)	4 (6.7%)	6 (6.1%)
ARID2	1 (2.7%)	0	1 (1.7%)	4 (4.0%)
APOB	3 (8.1%)	7 (9.7%)	7 (11.7%)	7 (7.1%)
NFE2L2	1 (2.7%)	0	2 (3.3%)	1 (1.0%)
AXIN1 mutation cluster&				
AXIN1	3 (8.1%)	4 (5.6%)	1 (1.7%)	1 (1.0%)
ARID1A	3 (8.1%)	6 (8.3%)	2 (3.3%)	4 (4.0%)
RPS6KA3	0	4 (5.6%)	1 (1.7%)	1 (1.0%)
TP53 mutation cluster&				
TP53	9 (34.3%)	27 (37.5%)	4 (6.7%)	20 (20.2%)
KEAP1	0	2 (2.8%)	2 (3.3%)	3 (3.0%)
TSC2	3 (8.1%)	4 (5.6%)	1 (1.7%)	1 (1.0%)
Mean CDRC expression z-score	−0.19	1.11	−0.60	0.03

CDRC, coordinate DNA repair cluster.

*Putative copy-number calls were determined using GISTIC 2.0. Values: −2 = homozygous deletion; −1 = heemizygous deletion; 0 = neutral/no change; 1 = gain; 2 = high-level amplification.

^#^For genes with multiple probes, methylation data was extracted from the probe with the strongest negative correlation between the methylation signal and the gene’s expression.

^#^The three major groups of associated alterations were identified using exome sequencing of HCC by Guichard *et al*.^20^.

**Table 2:** Univariate and multivariate analyses of prognostic parameters for overall survival and tumor-free survival in group 2 and group 3 patients.

**Prognostic parameter**	**Univariate analysis**			**Multivariate analysis**		
	**HR**	**95% CI**	**P value**	**HR**	**95% CI**	**P value**
**OS**						
Group 2 vs. Group 3	3.455	1.731–6.898	<0.001	2.989	1.431–6.243	0.004
Age (<60 vs. ≥60)	0.524	0.292–0.943	0.031			
Gender (male vs. female)	0.950	0.511–1.769	0.873			
History HCC risk factor (present vs. absent)	0.489	0.261–0.915	0.025			
Tumor grade (G3 + G4 vs. G1 + G2)	1.408	0.774–2.560	0.263			
Pathological stage (III + IV vs. I + II)	3.854	2.025–7.335	<0.001	3.290	1.712–6.320	<0.001
T classification (T3 + T4 vs. T1 + T2)	3.744	2.032–6.899	<0.001			
Vascular invasion (present vs. absent)	1.267	0.588–2.730	0.545			
TP53 mutation (present vs. absent)	1.170	0.593–2.308	0.650			
TFS						
Group 2 vs. Group 3	5.952	1.756–20.172	0.004	3.366	1.053–11.889	0.049
Age (<60 vs. ≥60)	0.493	0.205–1.187	0.115			
Gender (male vs. female)	0.973	0.388–2.439	0.954			
History HCC risk factor (present vs. absent)	0.322	0.127–0.817	0.017			
Tumor grade (G3 + G4 vs. G1 + G2)	1.183	0.477–2.934	0.717			
Pathological stage (III + IV vs. I + II)	6.288	2.311–17.111	<0.001			
T classification (T3 + T4 vs. T1 + T2)	6.768	2.488–18.411	<0.001	5.615	1.819–17.331	0.003
Vascular invasion (present vs. absent)	1.415	0.332–6.030	0.639			
TP53 mutation (present vs. absent)	1.526	0.639–3.647	0.342			

Group 2: highest expression of coordinate DNA repair cluster; Group 3: lowest expression of coordinate DNA repair cluster

**Table 3:** Characteristics of patient groups by MSH2 expression pattern.

	**Low group** (**n** = **41**)	**Moderate group** (**n** = **48**)	**High Group** (**n** = **31**)
Age (year)	58.9 ± 10.7	57.2 ± 10.1	61.2 ± 8.4
Gender			
Male (n, %)	33 (80.5%)	38 (79.2%)	27 (87.1%)
Female (n, %)	8 (19.5%)	10 (20.8%)	4 (12.9%)
Vital status			
Alive (n, %)	32 (78.0%)	38 (79.2%)	14 (45.2%)
Dead (n, %)	9 (22.0%)	10 (20.8%)	17 (54.8%)
Tumor grade			
G1 + G2 (n, %)	21 (51.2%)	27 (56.3%)	14 (45.2%)
G3 + G4 (n, %)	20 (48.8%)	21 (43.7%)	17 (54.8%)
AJCC TNM staging system (T)			
Tumor size			
T1 + T2 (n, %)	37 (90.2%)	33 (68.8%)	16 (51.6%)
T3 + T4 (n, %)	4 (9.8%)	15 (31.2%)	15 (48.4%)
AJCC TNM staging system (N)			
Lymph node involvement			
N0 (n, %)	38 (92.7%)	39 (81.2%)	24 (77.4%)
N1 (n, %)	2 (4.9%)	2 (4.2%)	2 (6.5%)
NX (n, %)	1 (2.4%)	7 (14.6%)	5 (16.1%)
AJCC TNM staging system (M)			
Metastasis status			
M0 (n, %)	40 (97.6%)	38 (79.1%)	22 (71.0%)
M1 (n, %)	0	3 (6.3%)	4 (12.9%)
MX (n, %)	1 (2.4%)	7 (14.6%)	5 (16.1%)
AJCC pathological stage			
I + II (n, %)	36 (87.8%)	33 (68.8%)	14 (45.2%)
III + IV (n, %)	5 (12.2%)	15 (31.2%)	17 (54.8%)
Vascular invasion			
yes (n, %)	6 (14.6%)	12 (25.0%)	9 (29.0%)
No (n, %)	35 (85.4%)	36 (75.0%)	22 (71.0%)
