# Supplementary material for: Prognostic value of DNA repair based stratification of hepatocellular carcinoma
Source: Sci Rep. 2016 May 13;6:25999. doi: 10.1038/srep25999 (PMC4867671; doi:10.1038/srep25999)
Supplement: Supplementary Information [file srep25999-s1.pdf]

Prognostic value of DNA repair based stratification of hepatocellular carcinoma

Zhuo Lin[1, 2]#, Shi-Hao Xu[3]#, Hai-Qing Wang[4], Yi-Jing Cai[1, 2], Li Ying[3], Mei Song[1, 2], Yu-Qun Wang[1, 2], Shan-Jie Du[1, 2], Ke-Qing Shi[1, 2]*, Meng-Tao Zhou[5]*

Supplementary Information

Supplementary Figure S1. Correlation amaong the 15 coordinate DNA repair cluster genes.

Pearson correlation between each gene was shown in the upperportion of Figure.

Supplementary Figure S2. Correlation between copy-number GISTIC and mRNA expression Z score of the coordinate DNA repair cluster genes.

The coloured represents significant negative correlation between copy-number GISTIC and mRNA expression Z score; The gray represents no correlation.

Supplementary Figure S3. Correlation between log2 copy-number values and mRNA expression Z score of the coordinate DNA repair cluster genes.

The coloured represents significant negative correlation between log2 copy-number values and mRNA expression Z score; The gray represents no correlation.

Supplementary Figure S4. The distribution of pathological parameter in each DNA repair molecular class of HCC patients.

HCC, hepatocellular carcinoma

Supplementary Figure S5. Correlation between DNA repair molecular classes and tumor-free survival in HCC patients was independent of tumor grade, pathological stage and vascular invasion.

(A) Comparisons of tumor-free survival in DNA repair molecular classes of HCC in

early tumor grade (G1-G2) cohort and in advanced tumor grade (G3-G4) cohort. (B) Comparisons of tumor-free survival in DNA repair molecular classes of HCC in early pathological stage (I-II) cohort and in advanced pathological stage (III-IV) cohort. (C) Comparisons of tumor-free survival in DNA repair molecular classes of HCC in early pathological T classification (T1-T2) cohort and in advanced pathological T classification (T3-T4) cohort. (D) Comparisons of tumor-free survival in DNA repair molecular classes of HCC in pathological no local lymph node metastasis cohort and in pathological no metastasis cohort. (E) Comparisons of tumor-free survival in DNA repair molecular classes of HCC in patients with or without vascular invasion.

P-values were calculated by log-rank test. Hazard ratios (95% Confidence Interval) and Log-rank Test were shown in the Tables.

HCC, hepatocellular carcinoma

Supplementary Figure S6. Correlation between DNA repair molecular classes and overall survival in HCC patients was independent of gender, age, and history risk factors.

(A) Comparisons of overall survival in DNA repair molecular classes of HCC in male cohort and in female cohort. (B) Comparisons of overall survival in DNA repair molecular classes of HCC in age < 60 cohort and in age ≥ 60 cohort. (C) Comparisons of overall survival in DNA repair molecular classes of HCC in without history risk factor cohort and in with history risk factor cohort.

P-values were calculated by log-rank test. Hazard ratios (95% Confidence Interval) and Log-rank Test were shown in the Tables.

HCC, hepatocellular carcinoma

Supplementary Figure S7. Correlation between DNA repair molecular classes and tumor-free survival in HCC patients was independent of gender, age, and history risk factors.

(A) Comparisons of tumor-free survival in DNA repair molecular classes of HCC in male cohort and in female cohort. (B) Comparisons of tumor-free survival in DNA repair molecular classes of HCC in age < 60 cohort and in age ≥ 60 cohort. (C) Comparisons of tumor-free survival in DNA repair molecular classes of HCC in without history risk factor cohort and in with history risk factor cohort.

P-values were calculated by log-rank test. Hazard ratios (95% Confidence Interval) and Log-rank Test were shown in the Tables.

HCC, hepatocellular carcinoma

Supplementary Figure S8. Correlation between DNA repair molecular classes and overall survival (A) and tumor-free survival (B) in HCC patients was independent of TP53 mutation.

P-values were calculated by log-rank test. Hazard ratios (95% Confidence Interval) and Log-rank Test were shown in the Tables.

HCC, hepatocellular carcinoma

Supplementary Figure S9. MSH2 mRNA expression in the groups identified by MSH2 immunochemistry.

Supplementary Figure S10. Correlation between MSH2 expression pattern and overall survival in the independent cohort.

(A) Correlation between MSH2 expression pattern and overall survival; (B) in early tumor grade (G1-G2) cohort; (C) in early pathological stage (I-II) cohort; (D) in early pathological T classification (T1-T2) cohort; (E) in pathological no local lymph node metastasis cohort; (F) in pathological no metastasis cohort; (G) in without vascular invasion cohort; (H) in male cohort; (I) in age ≥ 60 cohort.

P-values were calculated by log-rank test. Hazard ratios (95% Confidence Interval) and Log-rank Test were shown in the Tables.

HCC, hepatocellular carcinoma

Supplementary Figure S11. The histopathology representative images of HCC.

(A) G1; (B) G2; (C) G3; (D)G4

HCC, hepatocellular carcinoma

Supplementary Table S1. Human DNA repair genes related to hepatocellular carcinoma.

Supplementary Table S2. Genes within the coordinate DNA repair cluster.

Supplementary Table S3. Multivariate analysis of gene expression changes in key mutation groups.

Supplementary Table S4. List of all patients in the independent cohort.

Supplementary Table S5. Univariate and multivariate analyses of prognostic parameters for overall survival in MSH2 low expression group and high expression group.

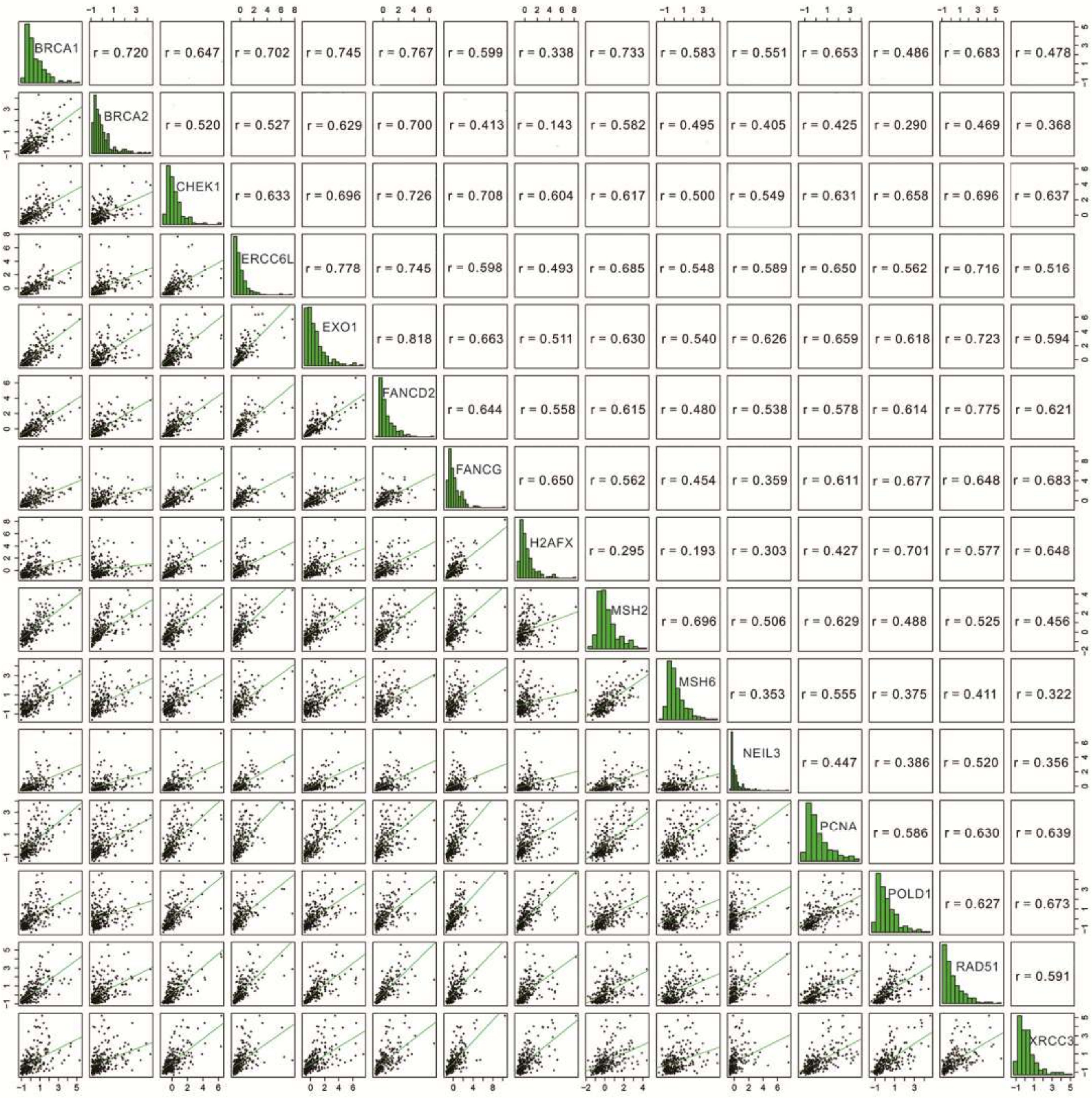

Supplementary Figure S1

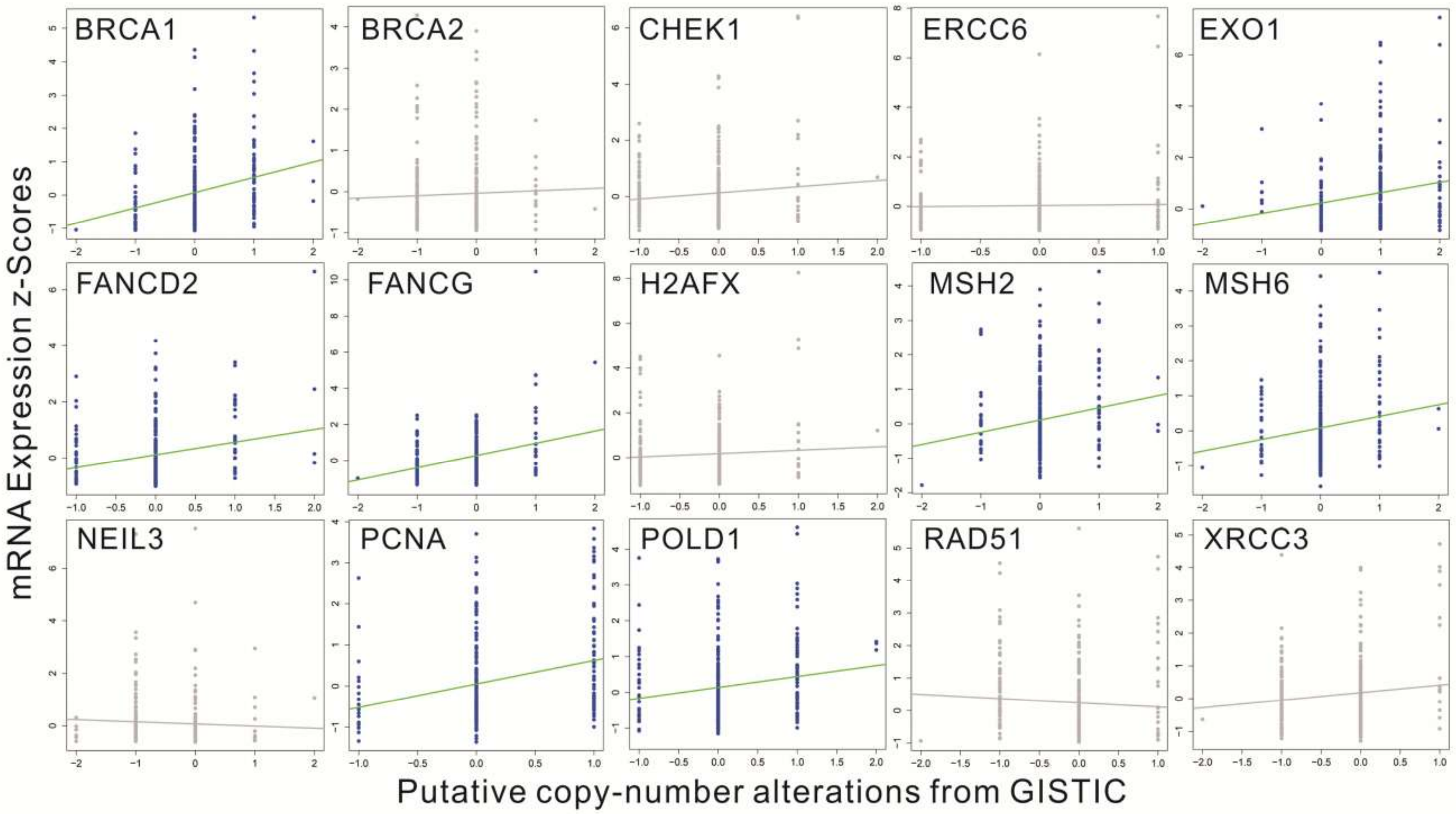

Supplementary Figure S2

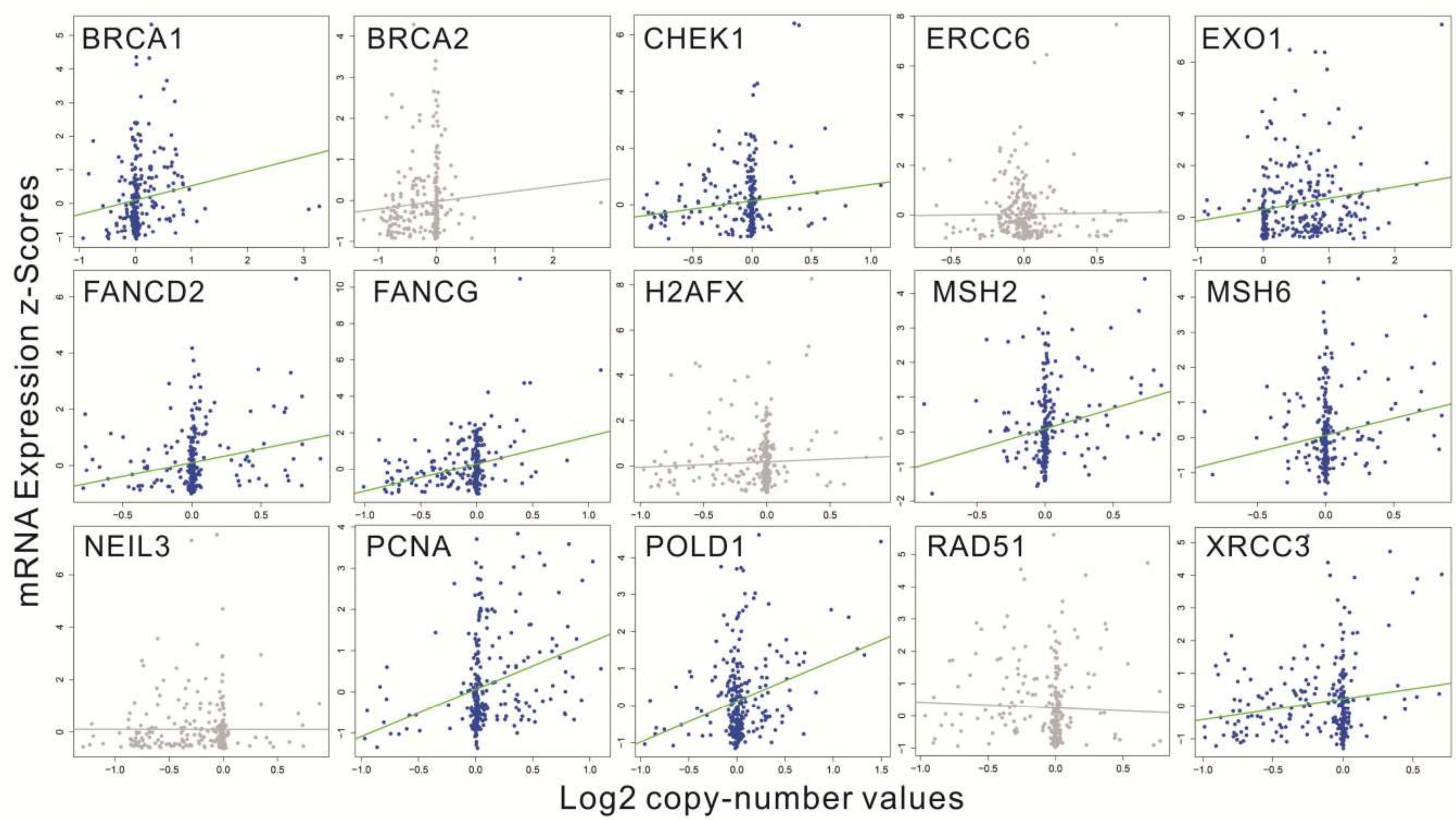

Supplementary Figure S3

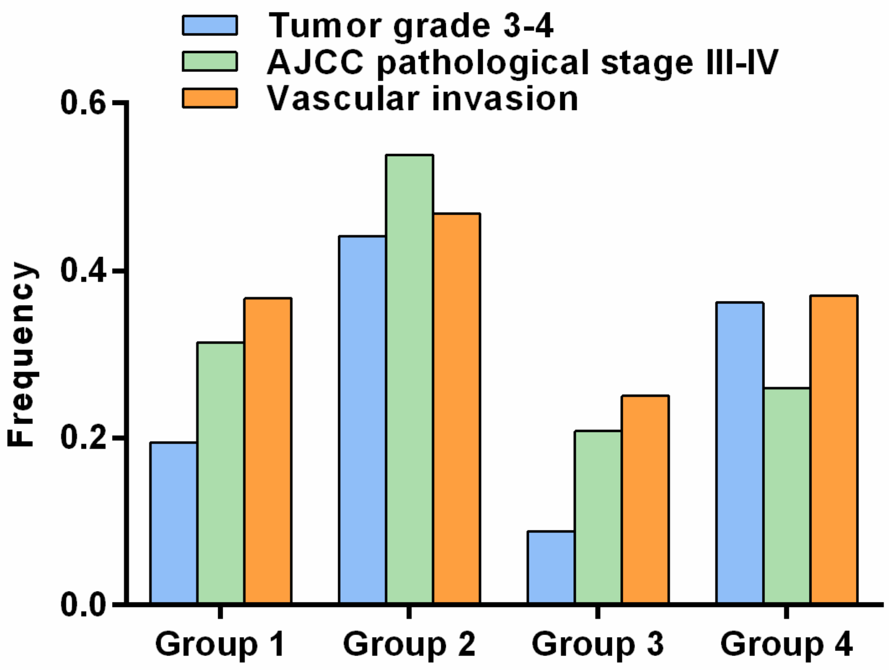

Supplementary Figure S4

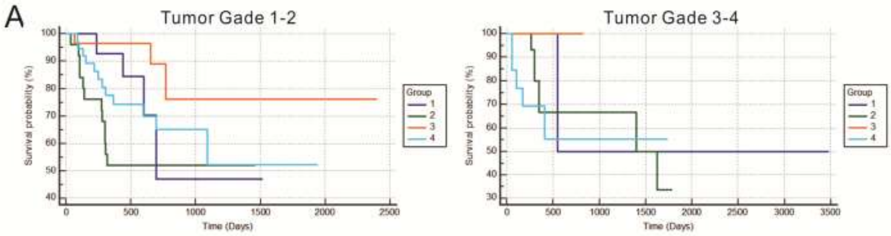

Hazard ratios (95% Confidence Interval) and Log-rank Test

		Tumor Gade 3-4			
	Group	1	2	3	4
Tumor Gade 1-2	1	-	1.97 (0.38 - 9.82)	-	2.29 (0.39 - 14.45)
			Log-rank P = 0.532		Log-rank P = 0.389
	2	0.50 (0.15 - 1.71)	-	-	1.15 (0.33 - 4.06)
		Log-rank P = 0.193			Log-rank P = 0.602
	3	2.93 (0.93 - 9.30)	**5.88 (2.14 - 16.18)**	-	-
		Log-rank P = 0.515	**Log-rank P = 0.0025**		
	4	0.89 (0.30 - 2.73)	1.75 (0.69 - 4.43)	**0.31 (0.11 - 0.86)**	-
		Log-rank P = 0.846	Log-rank P = 0.180	**Log-rank P = 0.0468**	

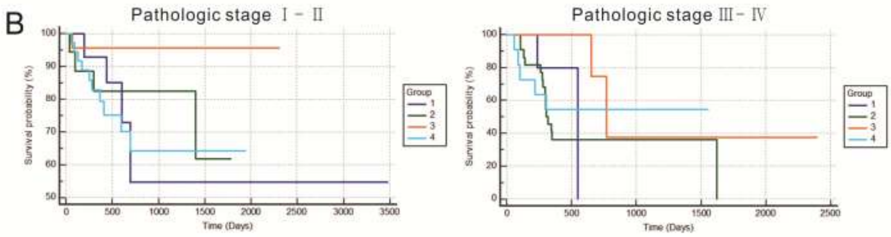

Hazard ratios (95% Confidence Interval) and Log-rank Test

		Pathologic stage Ⅲ. Ⅳ			
	Group	1	2	3	4
Pathologic stage Ⅰ - Ⅱ	1	-	1.34 (0.31 - 5.864)	0.43 (0.09 - 2.22)	0.94 (0.19 - 4.62)
			Log-rank P = 0.689	Log-rank P = 0.0523	Log-rank P = 0.822
	2	1.20 (0.27 - 5.28)	-	**0.32 (0.11 - 0.94)**	0.70 (0.25 - 1.92)
		Log-rank P = 0.767		**Log-rank P = 0.0482**	Log-rank P = 0.600
	3	6.31 (1.55 - 25.62)	5.28 (1.40 - 19.95)	-	2.15 (0.63 - 7.32)
		Log-rank P = 0.0594	Log-rank P = 0.134		Log-rank P = 0.494
	4	0.87 (0.23 - 3.27)	0.72 (0.21 - 2.54)	**0.14 (0.04 - 0.43)**	-
		Log-rank P = 0.715	Log-rank P = 0.464	**Log-rank P = 0.0281**	

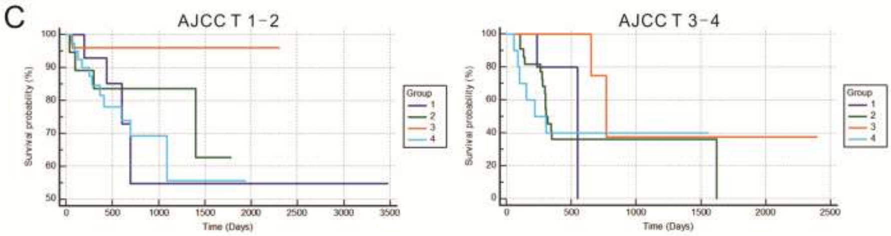

Hazard ratios (95% Confidence Interval) and Log-rank Test

		AJCC T 3-4			
	Group	1	2	3	4
AJCC T 1-2	1	-	1.36 (0.34 - 5.47)	0.44 (0.09 - 2.03)	1.61 (0.33 - 7.85)
			Log-rank P = 0.689	Log-rank P = 0.0523	Log-rank P = 0.369
	2	1.34 (0.34 - 5.22)	-	**0.32 (0.12 - 0.88)**	1.18 (0.40 - 3.49)
		Log-rank P = 0.685		**Log-rank P = 0.0482**	Log-rank P = 0.715
	3	**6.70 (1.62 - 27.64)**	4.97 (1.36 - 18.21)	-	3.66 (1.04 - 12.96)
		**Log-rank P = 0.0453**	Log-rank P = 0.127		Log-rank P = 0.155
	4	0.92 (0.24 - 3.46)	0.68 (0.21 - 2.26)	**0.14 (0.04 - 0.42)**	-
		Log-rank P = 0.810	Log-rank P = 0.386	**Log-rank P = 0.0283**	

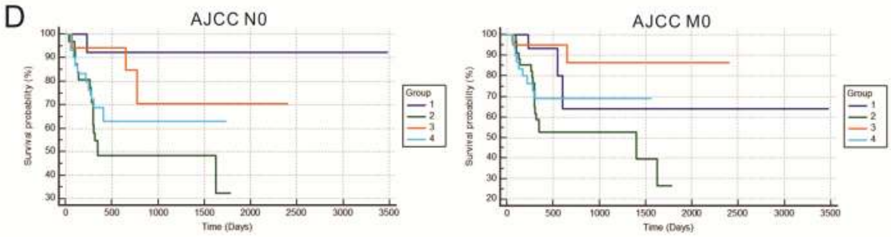

Hazard ratios (95% Confidence Interval) and Log-rank Test

		AJCC M0			
	Group	1	2	3	4
AJCC N0	1	-	2.80 (0.96 - 8.12)	0.41 (0.14 - 1.27)	1.77 (0.60 - 5.03)
			Log-rank P = 0.0805	Log-rank P = 0.992	Log-rank P = 0.413
	2	**0.13 (0.04 - 0.38)**	-	**0.15 (0.06 - 0.37)**	0.62 (0.26 - 1.48)
		**Log-rank P = 0.0186**		**Log-rank P = 0.0026**	Log-rank P = 0.322
	3	0.52 (0.16 - 1.68)	4.08 (1.56 - 10.67)	-	4.27 (1.62 - 11.24)
		Log-rank P = 0.206	Log-rank P = 0.117		Log-rank P = 0.0851
	4	0.20 (0.06 - 0.62)	1.60 (0.65 - 3.89)	0.39 (0.18 - 1.03)	-
		Log-rank P = 0.081	Log-rank P = 0.253	Log-rank P = 0.744	

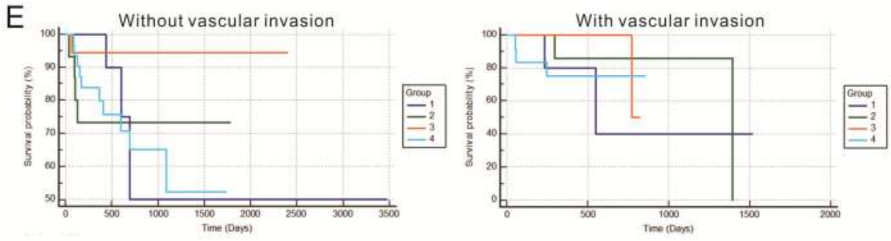

Hazard ratios (95% Confidence Interval) and Log-rank Test

		With vascular invasion			
	Group	1	2	3	4
Without vascular invasion	1	-	0.72 (0.09 - 5.66)	0.38 (0.05 - 3.01)	1.05 (0.13 - 8.13)
			Log-rank P = 0.824	Log-rank P = 0.199	Log-rank P = 0.720
	2	0.97 (0.23 - 4.08)	-	0.52 (0.08 - 3.51)	1.46 (0.22 - 9.47)
		Log-rank P = 0.942		Log-rank P = 0.962	Log-rank P = 0.497
	3	5.05 (1.12 - 22.67)	5.21 (1.29- 20.95)	-	2.79 (0.42 - 18.53)
		Log-rank P = 0.105	Log-rank P = 0.100		Log-rank P = 0.440
	4	0.80 (0.20 - 3.22)	0.82 (0.23 - 2.95)	**0.16 (0.05 - 0.51)**	-
		Log-rank P = 0.734	Log-rank P = 0.638	**Log-rank P = 0.0445**	

Supplementary Figure S5

A

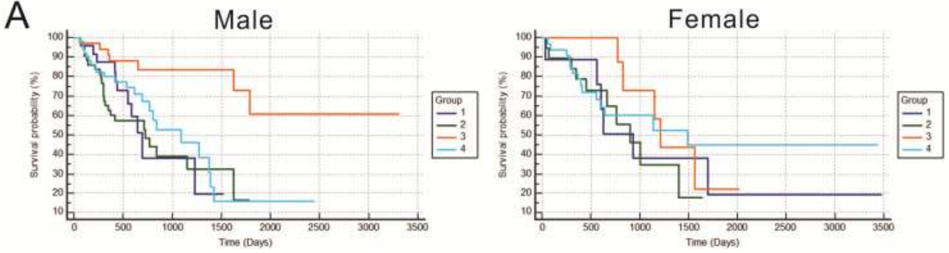

Hazard ratios (95% Confidence Interval) and Log-rank Test

		Female			
	**Group**	**1**	**2**	**3**	**4**
Male	**1**	-	1.01 (0.31 - 3.22)	0.52 (0.16 - 1.68)	0.68 (0.24 - 1.94)
			Log-rank P = 0.839	Log-rank P = 0.345	Log-rank P = 0.440
	**2**	0. 88 (0.38 - 2.04)	-	0.52 (0.19 - 1.43)	0.68 (0.28 - 162)
		Log-rank P = 0.714		Log-rank P = 0.170	Log-rank P = 0.443
	**3**	**3.66 (1.63 - 8.25)**	**4.15 (2.14 - 8.04)**	-	1.31 (0.55 - 3.16)
		**Log-rank P = 0.0036**	**Log-rank P = 0.0003**		Log-rank P = 0.565
	**4**	1.21 (0.54 - 2.72)	1.38 (0.71 - 2.65)	**0.33 (0.18 - 0.62)**	-
		Log-rank P = 0.521	Log-rank P = 0.335	**Log-rank P = 0.0051**	

B

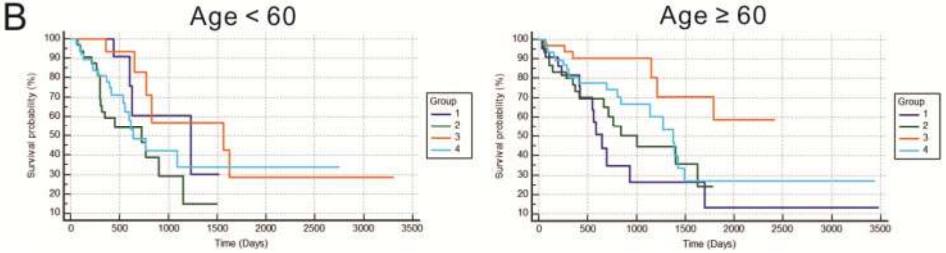

Hazard ratios (95% Confidence Interval) and Log-rank Test

		Age ≥ 60			
	**Group**	**1**	**2**	**3**	**4**
Age < 60	**1**	-	0.70 (0.28 - 1.78)	**0.21 (0.08 - 0.51)**	0.52 (0.22 - 1.25)
			Log-rank P = 0.367	**Log-rank P = 0.0003**	Log-rank P = 0.0702
	**2**	0.49 (0.14 - 1.67)	-	**0.29 (0.14 - 0.62)**	0.75 (0.36 - 1.53)
		Log-rank P = 0.178		**Log-rank P = 0.0054**	Log-rank P = 0.426
	**3**	1.63 (0.50 - 5.26)	**2.78 (1.21 - 6.39)**	-	**2.54 (1.30 - 4.94)**
		Log-rank P = 0.459	**Log-rank P = 0.0130**		**Log-rank P = 0.0373**
	**4**	1.49 (0.51 - 4.36)	1.52 (0.72 - 3.22)	0.55 (0.25 - 1.18)	
		Log-rank P = 0.536	Log-rank P = 0.310	Log-rank P = 0.190	

C

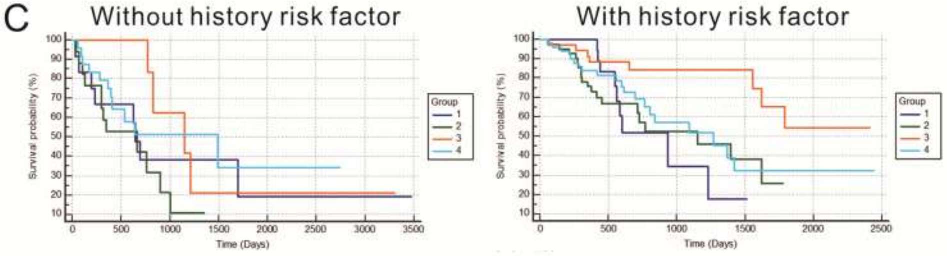

Hazard ratios (95% Confidence Interval) and Log-rank Test

		With history risk factor			
	**Group**	**1**	**2**	**3**	**4**
Without history risk factor	**1**	-	0.93 (0.36 - 2.39)	**0.32 (0.13 - 0.81)**	0.74 (0.30 - 1.83)
			Log-rank P = 0.900	**Log-rank P = 0.007**	Log-rank P = 0.385
	**2**	0.69 (0.24 - 1.95)	-	**0.35 (0.17 - 0.71)**	0.79 (0.40 - 1.59)
		Log-rank P = 0.464		**Log-rank P = 0.0064**	Log-rank P = 0.549
	**3**	2.26 (0.81 - 6.28)	**3.29 (1.19 - 9.08)**	-	**2.28 (1.18 - 4.40)**
		Log-rank P = 0.198	**Log-rank P = 0.0196**		**Log-rank P = 0.0412**
	**4**	1.38 (0.54 - 3.46)	2.01 (0.81 - 5.00)	0.61 (0.25 - 1.48)	-
		Log-rank P = 0.538	Log-rank P = 0.106	Log-rank P = 0.398	

Supplementary Figure S6

A

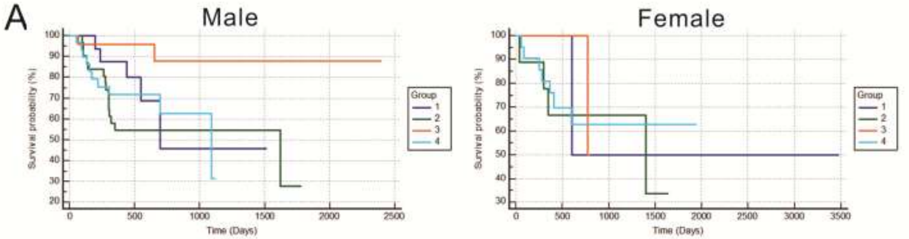

Hazard ratios (95% Confidence Interval) and Log-rank Test

		Female			
	**Group**	**1**	**2**	**3**	**4**
**Male**	**1**	-	1.49 (0.16 - 13.61)	0.48 (0.05 - 4.81)	1.11 (0.15 - 8.48)
			Log-rank P = 0.730	Log-rank P = 0.617	Log-rank P = 0.927
	**2**	0.62 (0.21 - 1.82)	-	0.32 (0.06 - 1.86)	0.75 (0.19 - 2.95)
		Log-rank P = 0.319		Log-rank P = 0.440	Log-rank P = 0.653
	**3**	**4.60 (1.56 - 13.54)**	**7.43 (3.01 - 18.38)**	-	2.30 (0.50 - 10.44)
		**Log-rank P = 0.0449**	**Log-rank P = 0.0018**		Log-rank P = 0.721
	**4**	0.80 (0.27 - 2.43)	1.30 (0.51 - 3.32)	**0.17 (0.07 - 0.45)**	-
		Log-rank P = 0.716	Log-rank P = 0.619	**Log-rank P = 0.0192**	

B

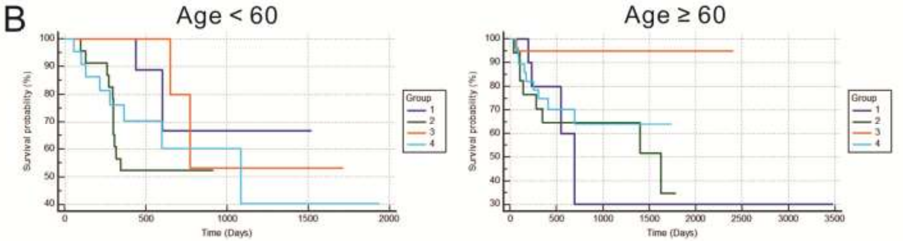

Hazard ratios (95% Confidence Interval) and Log-rank Test

		Age ≥ 60			
	**Group**	**1**	**2**	**3**	**4**
**Age < 60**	**1**	-	1.02 (0.24 - 4.38)	**0.11 (0.03 - 0.44)**	0.81 (0.20 - 3.26)
			Log-rank P = 0.988	**Log-rank P = 0.0096**	Log-rank P = 0.604
	**2**	**0.29 (0.09 - 0.99)**	-	**0.10 (0.03 - 0.33)**	0.80 (0.26 - 2.42)
		**Log-rank P = 0.0463**		**Log-rank P = 0.0126**	Log-rank P = 0673
	**3**	1.15 (0.31 - 4.28)	**4.00 (1.22 - 13.04)**	-	**7.68 (2.68 - 22.03)**
		Log-rank P = 0.854	**Log-rank P = 0.0435**		**Log-rank P = 0.0289**
	**4**	0.46 (0.14 - 1.52)	1.60 (0.56 - 4.56)	0.40 (0.13 - 1.26)	-
		Log-rank P = 0.307	Log-rank P = 0.393	Log-rank P = 0.259	

C

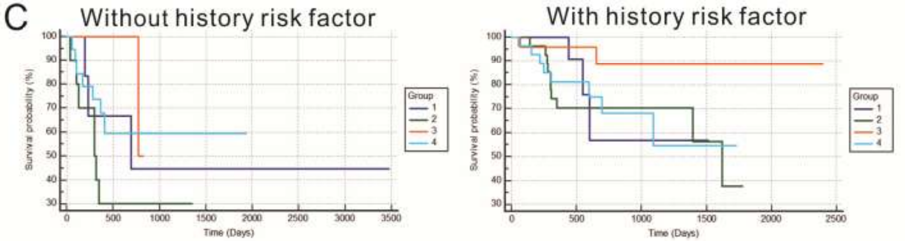

Hazard ratios (95% Confidence Interval) and Log-rank Test

		With history risk factor			
	**Group**	**1**	**2**	**3**	**4**
**Without history risk factor**	**1**	-	1.40 (0.35 - 5.59)	0.27 (0.07 - 1.08)	1.17 (0.29 - 4.73)
			Log-rank P = 0.542	Log-rank P = 0.111	Log-rank P = 0.929
	**2**	0.55 (0.11 - 2.73)	-	**0.19 (0.07 - 0.56)**	0.83 (0.29 - 2.47)
		Log-rank P = 0.404		**Log-rank P = 0.0224**	Log-rank P = 0.816
	**3**	3.82 (0.77 - 18.99)	**6.95 (1.55 - 31.17)**	-	4.30 (1.47 - 12.57)
		Log-rank P = 0.295	**Log-rank P = 0.0320**		Log-rank P = 0.0865
	**4**	1.37 (0.35 - 5.41)	2.48 (0.71 - 8.71)	0.36 (0.10 - 1.26)	-
		Log-rank P = 0.725	Log-rank P = 0.097	Log-rank P = 0.338	

Supplementary Figure S7

A

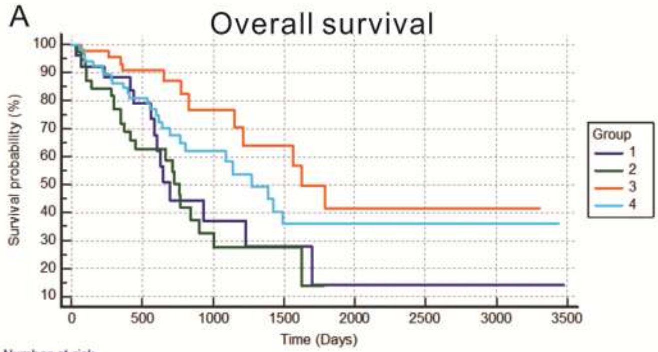

Number at risk

Group								
Group: 1	26	15	4	3	1	1	1	0
Group: 2	39	19	6	2	0	0	0	0
Group: 3	46	29	12	9	5	1	1	0
Group: 4	68	41	18	8	5	2	1	0

Hazard ratios (95% Confidence Interval) and Log-rank Test

Group	1	2	3	4
1	-	1.17 (0.52 - 2.67)<br>Log-rank P = 0.637	**0.37 (0.18 - 0.79)**<br>**Log-rank P = 0.0058**	0.63 (0.30 - 1.29)<br>Log-rank P = 0.176
2	0.85 (0.38 - 1.93)<br>Log-rank P = 0.637	-	**0.32 (0.16 - 0.62)**<br>**Log-rank P = 0.0004**	**0.53 (0.28 - 1.01)**<br>**Log-rank P = 0.0365**
3	**2.69 (1.27 - 5.71)**<br>**Log-rank P = 0.0058**	**3.16 (1.62 - 6.19)**<br>**Log-rank P = 0.0004**	-	1.69 (0.97 - 2.93)<br>Log-rank P = 0.116
4	1.59 (0.77 - 3.29)<br>Log-rank P = 0.176	**1.87 0.99 - 3.56)**<br>**Log-rank P = 0.0365**	0.69 (0.41 - 1.18)<br>Log-rank P = 0.116	-

B

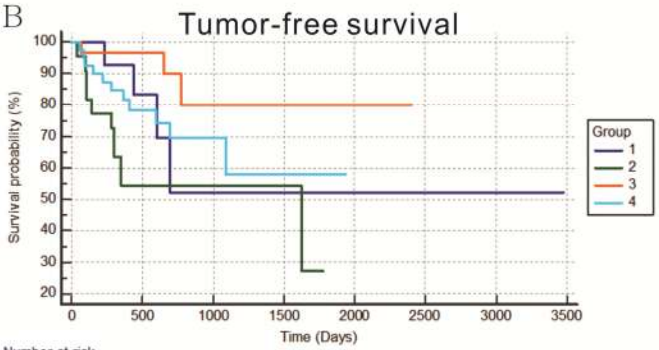

Number at risk

Group								
Group: 1	14	7	2	2	1	1	1	0
Group: 2	22	6	3	2	0	0	0	0
Group: 3	30	17	3	3	2	0	0	0
Group: 4	41	22	8	3	0	0	0	0

Hazard ratios (95% Confidence Interval) and Log-rank Test

Group	1	2	3	4
1	-	2.10 (0.56 - 7.89)<br>Log-rank P = 0.206	0.32 (0.10 - 1.07)<br>Log-rank P = 0.103	0.96 (0.30 - 3.04)<br>Log-rank P = 0.953
2	0.49 (0.14 - 1.68)<br>Log-rank P = 0.206	-	**0.15 (0.05 - 0.46)**<br>**Log-rank P = 0.0014**	0.46 (0.16 - 1.30)<br>Log-rank P = 0.0785
3	3.08 (0.93 - 10.15)<br>Log-rank P = 0.103	**6.47 (2.18 - 19.24)**<br>**Log-rank P = 0.0014**	-	2.95 (1.22 - 7.11)<br>Log-rank P = 0.0959
4	1.04 (0.32 - 3.32)<br>Log-rank P = 0.953	2.19 (0.77 - 6.27)<br>Log-rank P = 0.0785	0.34 (0.14 - 0.82)<br>Log-rank P = 0.0959	-

Supplementary Figure S8

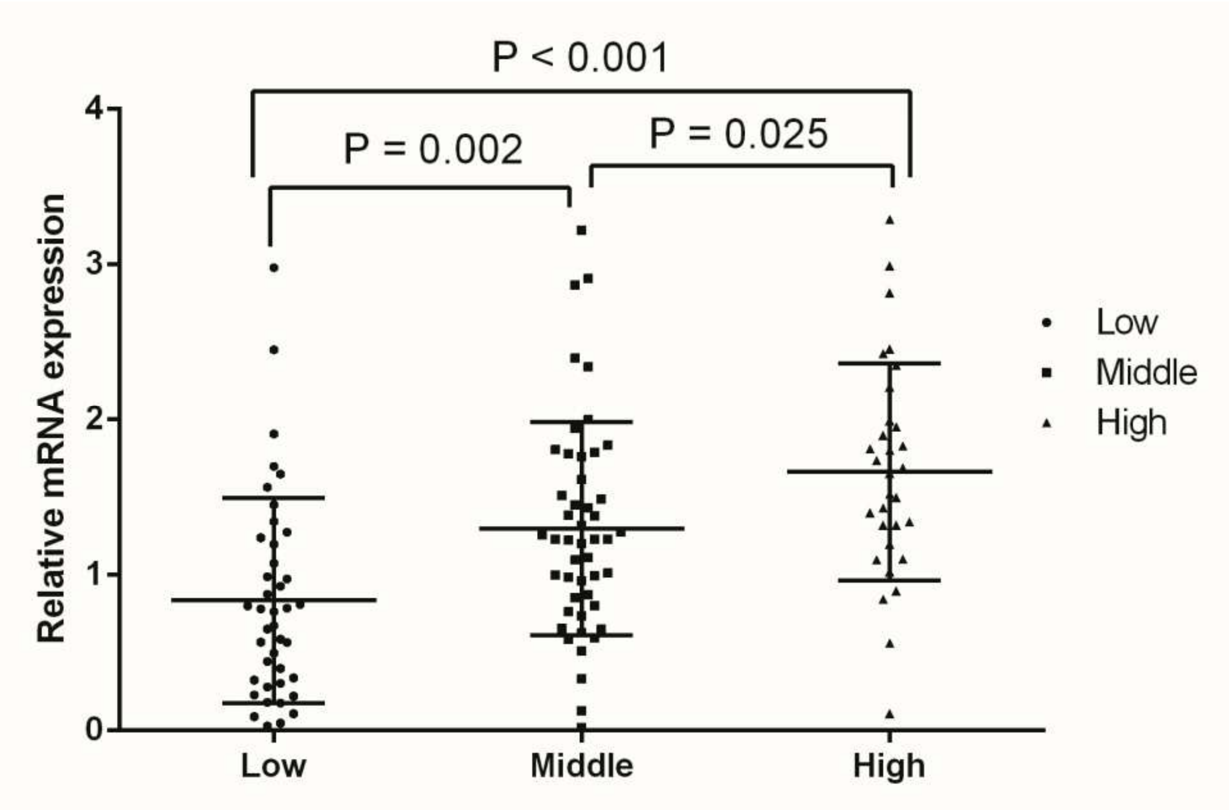

Supplementary Figure S9

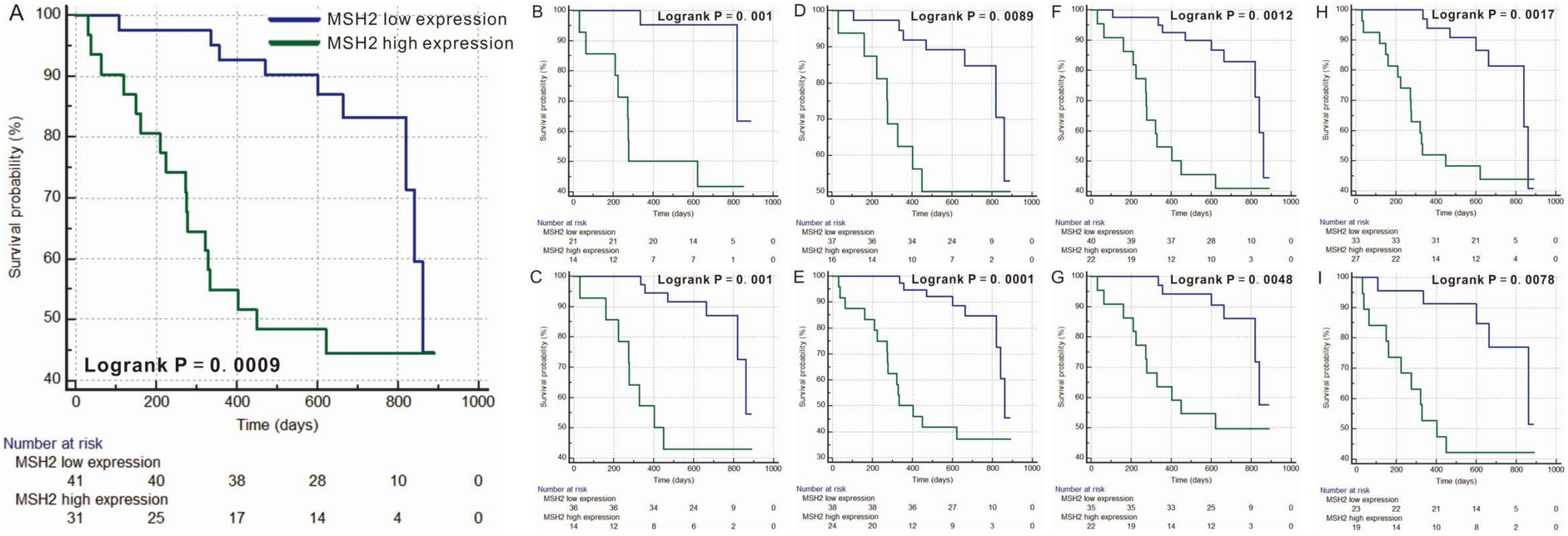

Supplementary Figure S10

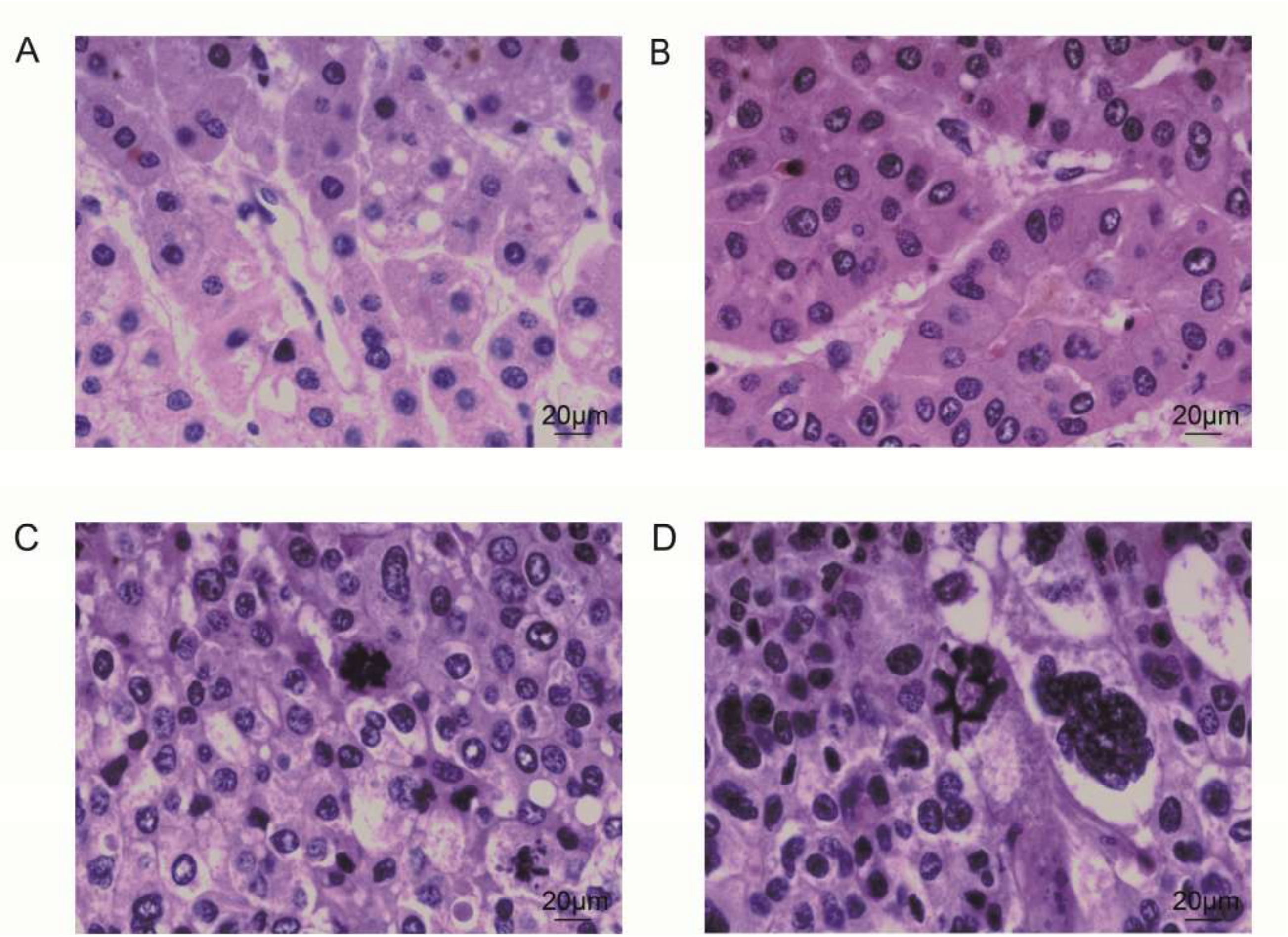

Supplementary Figure S11

Supplementary Table S1. Human DNA repair genes related to hepatocellular carcinoma.

Gene Symbol	Gene Name	Activity
**Base excision repair (BER)**		DNA glycosylases: major altered base released
MBD4	methyl-CpG binding domain protein 4	U or T opposite G at CpG sequences
TDG	thymine-DNA glycosylase	U, T or ethenoC opposite G
OGG1	8-oxoguanine DNA glycosylase	8-oxoG opposite C
NEIL3	nei endonuclease VIII-like 3	Removes oxidative products of pyrimidines
**Other BER and strand break joining factors**		
APEX1	APEX nuclease 1	AP endonuclease
XRCC1	X-ray repair complementing defective repair in Chinese hamster cells 1	LIG3 accessory factor
**Poly(ADP-ribose) polymerase (PARP) enzymes that bind to DNA**		
PARP1	poly (ADP-ribose) polymerase 1	Protects strand interruptions
**Direct reversal of damage**		
MGMT	O-6-methylguanine-DNA methyltransferase	O6-meG alkyltransferase
**Mismatch excision repair (MMR)**		
MSH2	mutS homolog 2	Mismatch (MSH2-MSH6) and loop (MSH2-MSH3) recognition
MSH3	mutS homolog 3	
MSH6	mutS homolog 6	
MLH1	mutL homolog 1	MutL homologs, forming heterodimer
PMS2	PMS2 postmeiotic segregation increased 2	
MLH3	mutL homolog 3	MutL homologs of unknown function
**Nucleotide excision repair (NER)**		
XPC	xeroderma pigmentosum, complementation group C	Binds DNA distortions
RAD23B	RAD23 homolog B	
XPA	xeroderma pigmentosum, complementation group A	Binds damaged DNA in preincision complex
DDB1	damage-specific DNA binding protein 1	Complex defective in XP group E
RPA2	replication protein A2	Binds DNA in preincision complex
**TFIIH**		
ERCC2 (XPD)	excision repair cross-complementing rodent repair deficiency, complementation group 2	5' to 3' DNA helicase
CDK7	cyclin-dependent kinase 7	Kinase subunits of TFIIH
CCNH	cyclin H	
ERCC5 (XPG)	excision repair cross-complementing rodent repair deficiency, complementation group 5	3' incision
ERCC1	excision repair cross-complementing rodent repair deficiency, complementation group 1	5' incision DNA binding subunit
**NER-related**		
ERCC8 (CSA)	excision repair cross-complementing rodent repair deficiency, complementation group 8	Cockayne syndrome and UV-Sensitive Syndrome; Needed for transcription-coupled NER

ERCC6 (CSB)	excision repair cross-complementing rodent repair deficiency, complementation group 6	
**Homologous recombination**		
RAD51	RAD51 recombinase	Homologous pairing
XRCC3	X-ray repair complementing defective repair in Chinese hamster cells 3	DNA break and crosslink repair
RAD52	RAD52 homolog	Accessory factors for recombination
BRCA1	breast cancer 1	Accessory factor for transcription and recombination, E3 Ubiquitin ligase
RAD50	RAD50 homolog	ATPase in complex with MRE11A, NBS1
MRE11A	MRE11 meiotic recombination 11 homolog A	3' exonuclease, defective in ATLD (ataxia-telangiectasia-like disorder)
NBN (NBS1)	nibrin	Mutated in Nijmegen breakage syndrome
MUS81	MUS81 structure-specific endonuclease subunit	Subunits of structure-specific DNA nuclease
**Fanconi anemia**		Tolerance and repair of DNA crosslinks and other adducts in DNA
FANCC	Fanconi anemia, complementation group C	FANCC
BRCA2 (FANCD1)	breast cancer 2	Cooperation with RAD51, essential function
FANCD2	Fanconi anemia, complementation group D2	target for monoubiquitination
FANCF	Fanconi anemia, complementation group F	
FANCG (XRCC9)	Fanconi anemia, complementation group G	
**Non-homologous end-joining**		
XRCC6 (Ku70)	X-ray repair complementing defective repair in Chinese hamster cells 6	DNA end binding subunit
XRCC5 (Ku80)	X-ray repair complementing defective repair in Chinese hamster cells 5	DNA end binding subunit
PRKDC	protein kinase, DNA-activated, catalytic polypeptide	DNA-dependent protein kinase catalytic subunit
LIG4	ligase IV	Ligase
XRCC4	X-ray repair complementing defective repair in Chinese hamster cells 4	Ligase accessory factor
**Modulation of nucleotide pools**		
DUT	deoxyuridine triphosphatase	dUTPase
RRM2B	ribonucleotide reductase M2 B	p53-inducible ribonucleotide reductase small subunit 2 homolog
**DNA polymerases (catalytic subunits)**		
POLD1	polymerase (DNA directed), delta 1	NER and MMR
PCNA	proliferating cell nuclear antigen	Sliding clamp for pol delta and pol epsilon
MAD2L2	MAD2 mitotic arrest deficient-like 2	DNA pol zeta subunit
**Editing and processing nucleases**		
EXO1	exonuclease 1	5' exonuclease
**Ubiquitination and modification**		

HLTF	helicase-like transcription factor	E3 ubiquitin ligase, SWI/SNF related, homolog of S. cerevisiae Rad5
**Chromatin Structure and Modification**		
H2AFX	H2A histone family, member X	Histone, phosphorylated after DNA damage
**Genes defective in diseases associated with sensitivity to DNA damaging agents**		
WRN	Werner syndrome, RecQ helicase-like	Werner syndrome helicase/3' - exonuclease
ATM	ataxia telangiectasia mutated	ataxia telangiectasia
**Other conserved DNA damage response genes**		
ATR	ataxia telangiectasia and Rad3 related	ATM- and PI-3K-like essential kinase
CHEK1	checkpoint kinase 1	Effector kinases
CHEK2	checkpoint kinase 2	
TP53	tumor protein p53	Regulation of the cell cycle
TP53BP1	tumor protein p53 binding protein 1	chromatin-binding checkpoint protein

Supplementary Table S2. Genes within the coordinate DNA repair cluster.

coordinate DNA repair gene
NEIL3
BRCA2
BRCA1
MSH2
MSH6
PCNA
CHEK1
FANCG
ERCC6
EXO1
FANCD2
RAD51
H2AFX
POLD1
XRCC3

These genes are presented in the order of the coordinate DNA repair cluster signature

Supplementary Table S3. Multivariate analysis of gene expression changes in key mutation groups.

Mutation	Gene Expression	Direction of change	P-value
TP53	BRCA1	Up	<0.001
	BRCA2	Up	<0.001
	CHEK1	Up	<0.001
	ERCC6	Up	<0.001
	EXO1	Up	<0.001
	FANCD2	Up	<0.001
	FANCG	Up	0.001
	H2AFX	Up	0.001
	MSH2	Up	<0.001
	MSH6	Up	0.004
	NEIL3	Up	0.002
	PCNA	Up	<0.001
	POLD1	Up	<0.001
	RAD51	Up	<0.001
	XRCC3	Up	<0.001
RPS6KA3	BRCA2	Up	0.004
	EXO1	Down	0.036
CTNNB1	ERCC6	Down	0.006
	FANCD2	Down	0.002
	H2AFX	Down	0.031
	MSH6	Down	0.001
	RAD51	Down	0.020
	XRCC3	Down	0.016
TSC2	NEIL3	Up	0.033

P values are derived from multivariate linear regression analysis.

Supplementary Table S4. Clinicopathological characteristics of patient in independent dataset.

Characteristics	Value
Age (year)	58.8 ± 10.0
Gender	
Male (n, %)	98 (81.7%)
Female (n, %)	22 (18.3%)
Vital status	
Alive (n, %)	84 (70.0%)
Dead (n, %)	36 (30.0%)
Tumor grade	
G1 + G2 (n, %)	62 (51.7%)
G3 + G4 (n, %)	58 (48.3%)
AJCC TNM staging system (T)	
Tumor size	
T1+T2 (n, %)	86 (71.7%)
T3+T4 (n, %)	34 (28.3%)
AJCC TNM staging system (N)	
Lymph node involvement	
N0 (n, %)	101 (84.2%)
N1 (n, %)	6 (5.0%)
NX (n, %)	13 (10.8%)
AJCC TNM staging system (M)	
Metastasis status	
M0 (n, %)	100 (83.4%)
M1 (n, %)	7 (5.8%)
MX (n, %)	13 (10.8%)
AJCC pathological stage	
I + II (n, %)	83 69.2%)
III + IV (n, %)	37 (30.8%)
Vascular invasion	
yes (n, %)	27 (22.5%)
No (n, %)	93 (77.5%)
MSH2 immunochemistry	
-	25 (20.8%)
+	16 (13.3%)
++	48 (40.0%)
+++	31 (25.9%)
Mean follow-up (Days)	612.0 ± 230.2

Supplementary Table S5. Univariate and multivariate analyses of prognostic parameters for overall survival in MSH2 low expression group and high expression group.

Prognostic parameter	Univariate analysis			Multivariate analysis		
	HR	95% CI	P value	HR	95% CI	P value
high vs. low	3.631	1.609 – 8.191	0.002	3.375	1.483 – 7.681	0.004
Age (< 60 vs. ≥ 60)	0.798	0.361 – 1.766	0.578			
Gender (male vs. female)	1.118	0.408 – 3.457	0.752			
Tumor grade (G3 + G4 vs. G1 + G2)	1.446	0.655 – 3.195	0.362			
Pathological stage (III + IV vs. I + II)	2.676	1.219 – 5.874	0.014			
T classification (T3 + T4 vs. T1 + T2)	2.703	1.225 – 5.965	0.014			
Vascular invasion (present vs. absent)	2.757	1.223 – 6.218	0.015	2.392	1.050 – 5.448	0.038
